# Targeted Epigenetic Activation of *CTLA4* in Haploinsufficiency Cellular Models by CRISPRa

**DOI:** 10.3390/biomedicines14040793

**Published:** 2026-03-31

**Authors:** Ekaterina Griazeva, Elizaveta I. Radion, Polina Kobyzeva, Natalia Ulasova, Elena Zelenova, Ekaterina Bolashova, Ivan Vladimirov, Valentin V. Makarov, Anton Keskinov, Vladimir Yudin

**Affiliations:** Federal State Budgetary Institution, Centre for Strategic Planning and Management of Biomedical Health Risks of the Federal Medical Biological Agency, Pogodinskaya Str., 10, Building 1, Moscow 119121, Russia

**Keywords:** *CTLA4*, haploinsufficiency, CRISPRa, off-target effects

## Abstract

**Background/Objectives**: *CTLA4* (Cytotoxic T-Lymphocyte Antigen 4) is a key immune checkpoint that plays a principal part in controlling T-cell activation and maintaining immune homeostasis. *CTLA4* haploinsufficiency (*CTLA4*^+/−^) results in severe autoimmune disorders and increased susceptibility to infections, often associated with dysregulated T cell activity. *CTLA4*^+/−^ patients experience life-threatening complications and exhibit moderate survival rates that can vary significantly, depending on the treatment of associated autoimmune phenotypes. Current therapeutic approaches primarily use immunosuppressives and monoclonal antibodies to modulate the immune response; however, their effectiveness and specificity are often limited. In this study, we aimed to develop a novel therapy approach for *CTLA4* haploinsufficiency utilizing the CRISPR activation system (CRISPRa) to enhance wild-type *CTLA4* allele expression. **Methods**: The CRISPR/Cas9 system has become a widely used method for precise genome editing, allowing for targeted gene regulation. We applied a CRISPRa-based strategy to induce endogenous *CTLA4* expression using appropriate cellular models and evaluated transcriptomic changes. **Results**: CRISPRa-mediated activation enabled increased *CTLA4* expression, suggesting the feasibility of restoring physiological expression levels. **Conclusions**: This approach may provide a basis for developing CRISPRa-based strategies to restore immune regulation and improve clinical outcomes in *CTLA4*^+/−^ patients.

## 1. Introduction

The *CTLA4* gene encodes a T cell surface receptor that acts as an immune repressor by competing with CD28 for binding of the common ligands CD80 and CD86 [1]. T cell activation requires two signals: the primary antigen-specific signal mediated by TCR binding to MHC-peptide complex, and a secondary co-stimulatory signal provided by the interaction between CD28 and its ligands, CD80/CD86, on antigen-presenting cells (APCs) [2]. These signals trigger downstream pathways such as P13K/AKT, RAS/ERK, and NF-kB, leading to T cell proliferation and cytokine production [3,4,5,6]. Normally, CD28-driven T cell activation stimulates elevated expression of *CTLA4*, forming an intricate feedback loop in immune response intensity regulation [7]. *CTLA4* is constitutively expressed in regulatory T cells (Tregs), mediating their immunosuppressive function [8]. In these cells, *CTLA4* plays a critical role in maintaining immune tolerance by modulating APC function, including downregulation of CD80/CD86 and induction of immunosuppressive pathways such as IDO [9,10,11]. Thus, *CTLA4* is a key regulator of immune homeostasis and prevents excessive immune responses.

*CTLA4* haploinsufficiency with immune infiltration (CHAI) is a severe, clinically heterogeneous disease characterized by prominent immune dysregulation [12]. Genetically, *CTLA4* haploinsufficiency can be a result of various mutations within the *CTLA4* gene body and promoter region [13]. In humans, *CTLA4* is located on chromosome 2 at position 2q33.2. The gene consists of four exons, which encode for a 223-amino acid protein. There are plethora of distinct mutations resulting in *CTLA4* malfunctioning and, in particular, to *CTLA4* haploinsufficiency. However, *CTLA4* heterozygous mutations may lead not just to haploinsufficiency of the wild-type allele but also to a dominant-negative effect caused by the mutant protein variant. In this regard, not all types of *CTLA4* genetic mutations should be considered as those leading to “clear haploinsufficiency”. Thus, the “clear haploinsufficiency” can be caused by early nonsense mutations, large early insertions/deletions, as well as alterations in the regulatory/promoter region, resulting in allele-specific transcription disruption. Patients with *CTLA4* haploinsufficiency present with a wide range of clinical symptoms, including autoimmune cytopenias, enteropathy, benign lymphoproliferation, recurrent respiratory tract infections, and malignancies [14]. Treatment options include immunosuppression with corticosteroids, mycophenolic acid, or calcineurin inhibitors. However, the toxicity of these drugs, as well as poor disease control, makes treatment problematic [15]. Very few data concerning pediatric cases [16,17] report a short-term disease control with fusion protein *CTLA4*-Fc (Abatacept). Strong immunosuppressive activity of abatacept can potentially lead to severe off-target effects in long-term treatment protocols, such as infections and malignant transformation [15,18,19]. Recently, a study on gene therapy for *CTLA4* insufficiency was published. The research conducted by Fox and colleagues utilized a homology-directed repair (HDR) gene editing strategy to insert *CTLA4* cDNA into the first intron of the *CTLA4* genomic locus. This approach resulted in restoration of *CTLA4*-regulated expression in CD4^+^ T cells [20]. However, the general limitation of dsDNA-break-based approaches is the danger of non-specific DNA cutting. Thus, a thorough off-target study must be performed when introducing such a protocol into clinical practice. This, in turn, would lead to a general increase in the therapy costs and impede the clinical implementation of the protocol.

To date, there is no clinically approved etiotropic therapy for *CTLA4* haploinsufficiency. The available therapeutic strategies can partially control disease manifestations by reducing immune activation or functionally compensating for *CTLA4* deficiency. However, they do not address the underlying genetic defects and are often associated with systemic immunosuppression, as well as increased susceptibility to infections. Hematopoietic stem cell transplantation has been explored as a potentially curative option, although it carries significant risks and is not universally applicable. Overall, currently available therapies are largely symptomatic and highlight the need for more precise and gene-targeted approaches [21,22]. In contrast to these approaches, CRISPRa-based strategies aim to directly restore gene expression, potentially providing a more targeted and physiologically relevant therapeutic effect compared to existing therapies. This supports a rationale for approaches aimed at restoring endogenous *CTLA4* expression rather than only controlling downstream immune dysregulation.

Since the molecular basis of haploinsufficiency is the insufficiency of a single wild-type allele copy to produce a wild-type phenotype, one possible therapeutic strategy may be to double the wild-type allele expression. This is possible with the use of CRISPR-based platforms. For almost 15 years, CRISPR-based technologies have been used in a wide range of genetic editing protocols, gene expression modulation, diagnostics, and pathogen detection [23,24,25,26]. In particular, CRISPR activation (CRISPRa) utilizes a catalytically inactive Cas9 (dCas9) fused to transcriptional activators to enable targeted upregulation of gene expression without introducing double-strand DNA breaks [27]. Guide RNAs (gRNAs) direct the dCas9-activator complex to regulatory regions, typically in close proximity to the transcriptional start site (TSS), where it promotes the recruitment of the transcriptional machinery and enhances transcription of the target gene [28]. Several studies have shown encouraging results for the CRISPR-based activation system in vitro and in vivo [29,30]. Indeed, in 2019, a study by Matharu and colleagues demonstrated CRISPRa-mediated rescue of obesity caused by *Sim1* haploinsufficiency in a mouse model [31]. Next, two independent studies conducted in 2020 reported an enhanced *Scn1a* expression in a haploinsufficiency mouse Dravet Syndrome model by dCas9-VP160 [32,33]. More recent examples show the usage of CRISPRa in the rescue of TTNtv-related functional deficits in dilated cardiomyopathy [34] and in the activation of EYA1 protein expression in BOR/BO syndrome [35]. These studies demonstrate the potential of CRISPRa-based approaches in the therapy of haploinsufficiency-related diseases. Despite promising potential and effective CRISPR-based approaches, there are potential risks of non-specific editing, so-called off-target effects. In this case, the gRNA sequence can have high resemblance to various genomic loci, and in turn, can erroneously attract Cas nucleases to these unintended sites. This unintended recognition can lead to DNA cutting at the spurious sites in the case of using functionally active Cas nucleases, or to non-specific editing of gene activity in the case of dCas9. As a result, DNA mutations, as well as unwanted gene expression changes, can be introduced, leading to deleterious effects. To minimize risks of off-target effects for CRISPR-based approaches, a comprehensive off-target analysis should be performed for each CRISPR-based protocol. This includes gRNA optimization and preclinical off-target validation, as well as genomic rearrangement assessment [36]. Moreover, for clinical use, the CRISPR-based approaches should also be tested in vitro for tumorgenicity, as well as for oncogenicity in vivo.

In the present study, we utilized CRISPRa to improve *CTLA4* expression levels in haploinsufficiency. For this, we first created *CTLA4* haploinsufficiency models in HEK293T and Jurkat cells and then tested if dCas9-VPR can restore *CTLA4* expression. We demonstrate the effectiveness of dCas9-VPR in the enhancement of *CTLA4* expression and protein abundance. Also, to address possible off-target effects, we conducted a thorough off-target assessment by transcriptomic analyses upon our CRISPRa protocol. Our findings suggest that CRISPRa may represent a promising approach for modulating gene expression in haploinsufficiency-associated disorders.

## 2. Materials and Methods

### 2.1. CTLA4 Haploinsufficiency Cell Models Construction

To delete the whole *CTLA4* ORF, two guide RNAs (gRNAs) in close proximity to the 5′-end of the *CTLA4* ORF and one gRNA in the 3′-end of the *CTLA4* ORF were cloned into pMA vectors under the U6 promoter. The gRNA sequences were designed using the Synthego online gRNA design tool: “https://www.synthego.com/products/bioinformatics/crispr-design-tool (accessed on 21 December 2022)”. The gRNA sequences and adjacent PAM sites are presented in Table 1.

**Figure A1:**
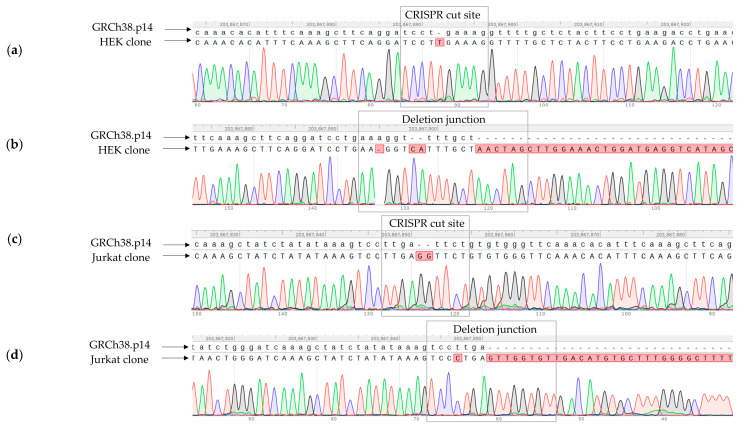
Sanger sequencing validation of *CTLA4* PCR amplicons. Representative Sanger sequencing chromatograms of PCR products obtained using primer pair 1 (5′ flanking) (**a**,**c**) and primer pair 2 (*CTLA4* deletion-junction PCR) (**b**,**d**), as schematically shown in Figure 1c,d. PCR chromatograms (**a**,**b**) correspond to HEK293T derivatives, and PCR chromatograms (**c**,**d**) correspond to Jurkat derivatives. Sequencing reads were aligned to the human reference genome (GRCh38) to confirm amplicon identity and correct the genomic origin of the PCR products across the targeted *CTLA4* locus. Colors correspond to nucleotide identity in Sanger sequencing chromatograms (A—green, T—red, C—blue, G—black).

gRNA-containing vectors were delivered into HEK293T and Jurkat cells along with Cas9-GFP plasmid by lipofection/electroporation, respectively. Then, in 72 h after plasmid delivery, the GFP-positive cells were sorted out and cloned by limiting dilutions. The monoclonal cell derivatives were screened for the presence of the whole ORF *CTLA4* deletion by PCR. The screening primers are presented in Table 2. The specific PCR products were gel-purified and subjected to Sanger sequencing using the corresponding primers. The raw sequencing chromatograms were aligned to the reference *CTLA4* genomic sequence (GRCh38) to confirm the amplicon identity (Appendix A, Figure A1).

### 2.2. CTLA4 Epigenetic Activation

gRNAs for CRISPR-mediated activation were designed to be located 500 bp upstream of *CTLA4* TSS with CHOPCHOP v3 software: “https://chopchop.cbu.uib.no/ (accessed on 26 May 2023)” and verified online with flyrnai.org. Candidate gRNA sites were first identified within the promoter region upstream of the TSS and then filtered based on PAM availability, predicted on-target efficiency, and minimal off-target potential. Next, the selected 10 gRNAs were designed and cloned into the pMA vector under the U6 promoter, and then all 10 gRNAs containing cassettes were assembled into 1 vector by Golden Gate cloning and verified by Sanger sequencing. The sequences of corresponding gRNAs, as well as adjacent PAM sites, are shown in Table 3.

**Figure A2:**
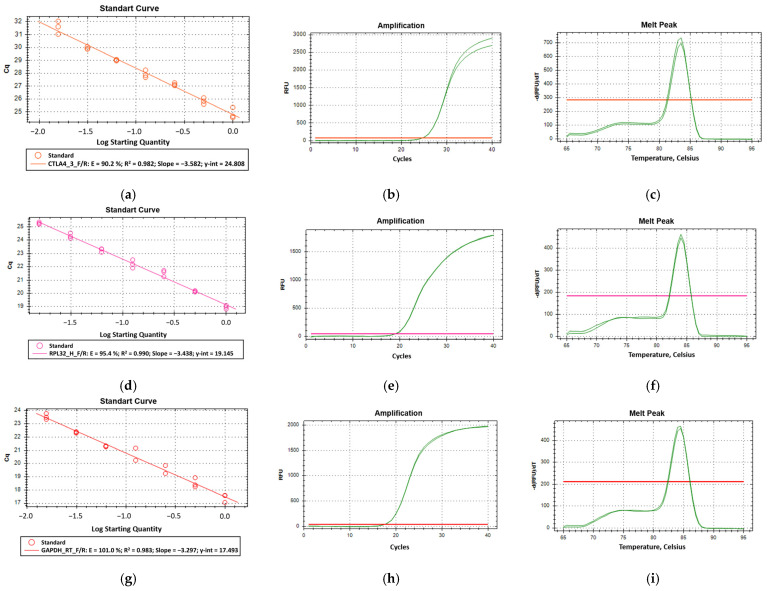
Validation of qPCR assays for *CTLA4*, *RPL32*, and *GAPDH* genes. Standard curves (**left panels**), amplification plots (**middle panels**), and melting curves (**right panels**) for (**a**–**c**) *CTLA4*, (**d**–**f**) *RPL32*, and (**g**–**i**) *GAPDH*. Flat lines in amplifications as well as melt curve plots represent threshold levels.

The dCas9-VPR-containing plasmid, as well as the plasmid bearing 10 activating gRNAs, was delivered into HEK293T and Jurkat cells by lipofection/electroporation. The cells were collected 72 h post-transfection. Then, to assess the efficiency of *CTLA4* activation, the total RNA fraction was isolated from cell samples, followed by RT-qPCR. For HEK293T and Jurkat cell lines, *CTLA4* expression was normalized to the *RPL32* gene. For direct comparison of *CTLA4* expression levels between Jurkat-derived lines and primary T cells, normalization was performed using the geometric mean of two housekeeping genes (*RPL32* and *GAPDH*). The corresponding primer sequences are shown in Table 4. Primer specificity and efficiency were validated using twofold serial dilutions of pooled cDNA, and the resulting slopes ranged from −3.297 to −3.582 (90–101% efficiency, R2 ≥ 0.98) (Appendix A, Table A1, and Figure A2).

### 2.3. Surface and Intracellular Staining

Jurkat cells were analyzed for CD69 surface expression, as well as *CTLA4* intracellular expression, using either anti-CD69 eFluor450 (clone FN50, Invitrogen, Carlsbad, CA, USA) or anti-CD152 PE (clone REA1003, Miltenyi Biotec, Bergisch Gladbach, Germany) monoclonal antibody. Cells were collected at 16 h, 18 h, 24 h, 48 h, or 72 h after electroporation and incubated for 30 min on ice in PEB buffer with the anti-CD69 antibody. After incubation, the antibody was washed out, and the cells were fixed and permeabilized, followed by intracellular staining with anti-*CTLA4* antibody using the Intracellular Fixation and Permeabilization Buffer Set (Invitrogen, Carlsbad, CA, USA) according to the manufacturer’s instructions.

Freshly isolated human peripheral blood mononuclear cells (PBMCs) were stained with anti-CD3 FITC (clone UCHT1, Beckman Coulter, Marseille, France), anti-CD4 PC5 (clone 13B8.2, Beckman Coulter, Marseille, France), and anti-CD25 Brilliant Violet 650 (clone BC96, Biolegend, San Diego, CA, USA) or anti-CD25 Superbright^TM^600 (clone BC96, Invitrogen, Carlsbad, CA, USA). Antibodies used during MACS separation (Miltenyi Biotec, Bergisch Gladbach, Germany) were applied according to the manufacturer’s instructions. On day 10 of expansion, the T cell viability was assessed with the Zombie Aqua™ Fixable Viability Kit (Biolegend, San Diego, CA, USA), followed by the phenotype verification with both intracellular *CTLA4* staining and surface CD4 staining. Flow cytometry analysis was performed using a CytoflexLX flow cytometer and CytExpert Software (v2.4; Beckman Coulter, Brea, CA, USA).

### 2.4. Cell Culture

Jurkat and HEK293T cell lines were obtained from the Russian Collection of Cell Cultures (RCCC). Jurkat cells were cultured in RPMI 1640 medium (PanEco, Moscow, Russia) supplemented with 10% fetal bovine serum (FBS) (Capricorn Scientific, Ebsdorfergrund, Germany), L-glutamine (2 mM), penicillin (100 U/mL), and streptomycin (100 μg/mL) (PanEco, Moscow, Russia). HEK293T cells were cultured in DMEM (PanEco, Moscow, Russia) with the same supplementation.

To isolate the CD4^+^CD25^−^ T cell subset, PBMCs were purified using Ficoll (PanEco, Moscow, Russia) gradient centrifugation, followed by magnetic-activated cell sorting using the CD4^+^CD25^+^ Regulatory T Cell Isolation Kit (Milteniy Biotec, Bergisch Gladbach, Germany). Next, the purified cells were cultured in TexMACS™ Medium (Milteniy Biotec, Bergisch Gladbach, Germany) supplemented with IL-2 (100 U/mL) (Gibco, Carlsbad, CA, USA), TGF-β (5 ng/mL) (Gibco, Carlsbad, CA, USA), and Rapamycin (100 ng/mL) (Sigma-Aldrich, St. Louis, MO, USA) at a density 1–2 × 10^6^ cells/mL and activated by TransAct™ (Miltenyi Biotec, Bergisch Gladbach, Germany) according to manufacturer’s instructions. On day 10 of expansion, cells were collected for flow cytometry and RT-qPCR analysis. All cell lines were cultured at 37 °C and 5% CO_2_.

### 2.5. Transcriptome Analysis and Libraries Preparation

RNA-seq libraries were prepared using the NEBNext Ultra II Directional RNA Library Prep Kit (New England Biolabs, Ipswich, MA, USA). For this purpose, RNA samples with a RIN value of at least 7 were used. The QC of the prepared libraries was carried out with a Qubit 4 Fluorimeter (Thermo Fisher Scientific, Waltham, MA, USA) and a TapeStation 4200 system (Agilent Technologies, Santa Clara, CA, USA). The selected fragment length was 300 bp. Sequencing was performed using NovaSeq 6000 (Illumina, San Diego, CA, USA), with the coverage of at least 30 million reads per sample.

### 2.6. Statistical and Bioinformatics Processing Methods

All statistical analyses were performed using GraphPad Prism 8.3.0 (GraphPad Software, San Diego, CA, USA). The data were presented as individual biological replicates with mean ± standard deviation (SD).

For RT-qPCR experiments, the ΔCt and ΔΔCt values were subjected to statistical analysis. For this, the comparisons between two independent groups were performed using unpaired two-tailed *t*-tests for normal distribution, or Mann–Whitney U tests otherwise. In case of comparisons involving more than two groups, either the one-way ANOVA with Tukey’s multiple comparison or Kruskal–Wallis with Dunn’s post hoc test was used for approximately normally distributed data or non-parametric data, respectively. For RT-qPCR data visualization, the relative expression was transformed to 2^−ΔCt^ or 2^−ΔΔCt^ and was presented as fold change (FC).

For flow cytometry experiments, data were presented either as the percentage of marker-positive cells or as geometric mean fluorescence intensity ratios (geoMFI/geoMFI). The time-course experiments assessing the CD69/*CTLA4* expression upon either PMA/ionomycin or CRISPRa stimulation were analyzed using multiple unpaired *t*-tests comparing stimulated vs control groups at each time point. The *p*-values were corrected for multiple testing using the Benjamini–Krieger–Yekutieli false discovery rate (FDR) method (Q = 5%).

A *p*-value < 0.05 was considered statistically significant. Levels of significance are indicated in the figures as *, *p* < 0.05; **, *p* < 0.01; ***, *p* < 0.001; ****, *p* < 0.0001; and ns, not significant.

Transcriptome analysis was carried out by Salmon 1.10.1 [37]. The Gencode transcriptome assembly GRCh38.p13 was used as a reference. Sequencing quality assessment was executed using fastQC 0.11.9 and MultiQC 1.13. Differential expression analysis was done by DESEQ2 R software (v1.39.8). The padj values were used to determine the significance level. Pathway analysis of differentially expressed genes was done using the ORA (overrepresented analysis) and GSEA (gene set enrichment analysis). For ORA, a list of genes with padj < 0.05 was used. For GSEA, a list of all significant genes corrected for multiple comparisons was used for gene lists of more than 100 genes, or the 100 most significant genes for gene lists of less than 100. The log_2_FC was used as the effect size variable in GSEA.

## 3. Results

### 3.1. CTLA4 Haploinsufficiency Model Construction

**Figure 1:**
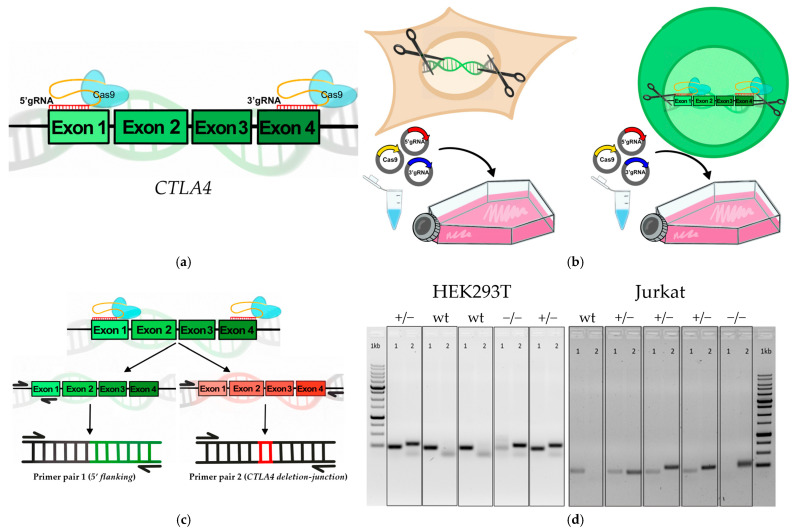
Generation of *CTLA4*^+/−^ and *CTLA4*^−/−^ cell lines. (**a**) Schematic representation of the *CTLA4* gene structure. Shown are gRNAs used for CRISPR KO; (**b**,**c**) schematic representation of the CRISPR KO protocol on HEK293T and Jurkat cell lines; (**d**) PCR screening of monoclonal HEK293T and Jurkat cell lines. The numbers on the top of the gel indicate the numbers of the primer pairs shown on the panel (**c**). The primer pair 1 (5′ flanking) allows detection of the intact allele, whereas the primer pair 2 (*CTLA4* deletion-junction) is specific to the whole *CTLA4* ORF deletion. Together, these PCR assays allow discrimination of *CTLA4*^+/+^ and *CTLA4*^+/−^, as well as *CTLA4*^−/−^ derivatives.

**Figure A3:**
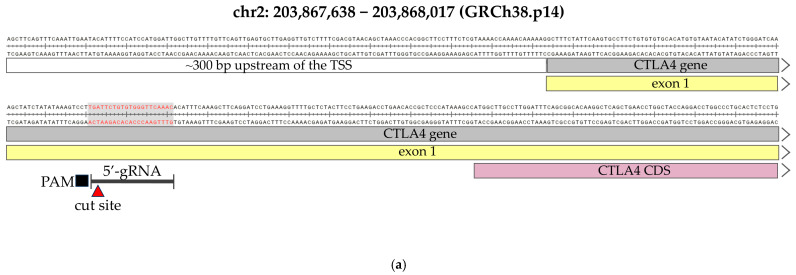

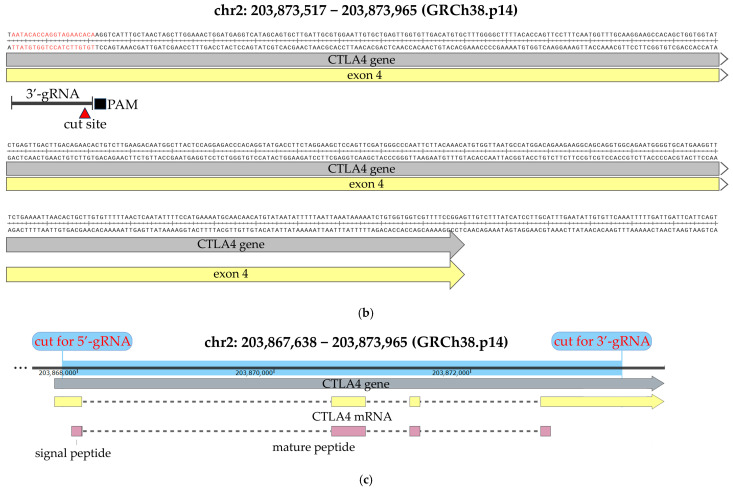
Schematic representation of the disposition of (**a**) 5′- and (**b**) 3′ gRNAs used to generate *CTLA4* KO. gRNA sequences are highlighted in red, and PAMs and cut sites are shown as black rectangles and red triangles, respectively; (**c**) disposition of both 5′ and 3′ gRNAs inside the *CTLA4* gene. The grey bar represents the *CTLA4* gene body; the yellow bars represent the *CTLA4* gene exons; the pink bars represent the *CTLA4* coding region.

To have a framework for assessing CRISPR-based activation, we modeled *CTLA4* haploinsufficiency in HEK293T and Jurkat cell lines. For this, we used the HEK293T cell line and aimed to cut out one or both *CTLA4* alleles to generate haplo- (*CTLA4*^+/−^) and total null (*CTLA4* KO and *CTLA4*^−/−^) genotypes. For *CTLA4* KO, we designed gRNAs flanking the *CTLA4* ORF. The cut site for 5′ gRNA was located at position −80/−121 bp, whereas the 3′ gRNA cut site was located 431 bp before the 3′end of exon 4 (Figure 1a–c; Appendix A, Figure A3). The gRNAs were cloned into the pMA-U6 vector, and then both the pSpCas9(BB)-2A-GFP and gRNA-containing vectors were transfected into HEK293T. Then, since the Cas9 protein is fused with GFP in the pSpCas9(BB)-2A-GFP vector, we sorted out the GFP-positive cells and cloned them by limiting dilutions. This resulted in a series of monoclonal cell derivatives that were expanded until full confluency. The final monoclonal cell lines were screened for complete *CTLA4* ORF deletion, as well as for the presence of an intact coding allele. We finally selected two *CTLA4*^+/−^ and one *CTLA4*^−/−^ cell derivatives (Figure 1d). Sanger sequencing analysis confirmed that the remaining allele retained an intact ORF without coding sequence disruption (Appendix A, Figure A1).

Next, we used Jurkat cells, as the closest cell model to T cells. As in HEK293T, we aimed to generate both haploinsufficiency and total *CTLA4* null cell lines. This resulted in one *CTLA4*^+/−^ and one *CTLA4*^−/−^ monoclonal line. Thus, we generated *CTLA4* haploinsufficiency cell models to further address the possibility of using the CRISPRa platform to selectively activate the remaining intact allele (Figure 1d). Sanger sequencing analysis further confirmed that the remaining allele retained an intact ORF without coding sequence disruption (Appendix A, Figure A1).

### 3.2. CTLA4 Epigenetic Activation with CRISPRa

**Figure 2:**
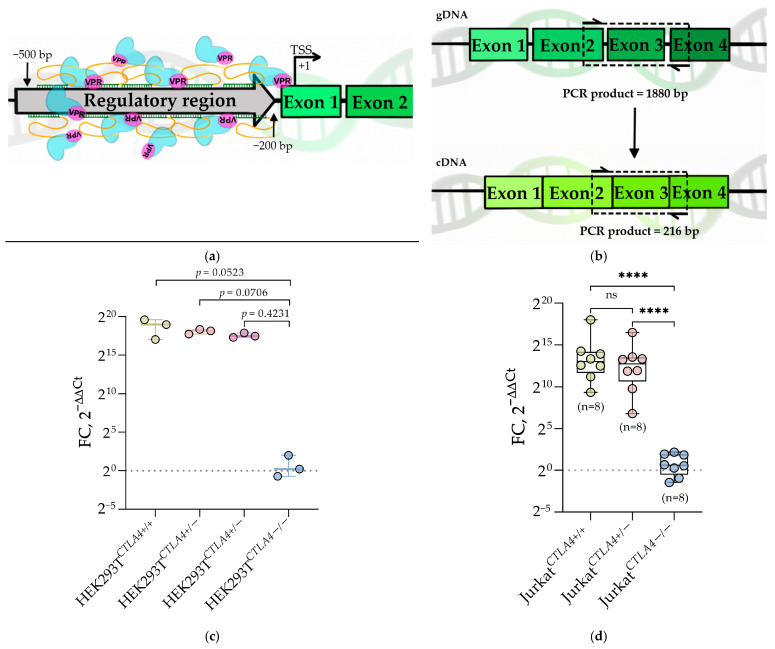
CRISPRa *CTLA4* expression activation. (**a**) Schematic representation of the activating gRNA disposition relative to *CTLA4* TSS; (**b**) schematic representation of the RT-qPCR primers position that was used to quantify *CTLA4* transcript induction; (**c**) *CTLA4* activation in HEK293^*TCTLA4*+/+^, HEK293^*TCTLA4*+/−^, and HEK293^*TCTLA4*−/−^ cells upon CRISPRa. HEK293^*TCTLA4*+/−^ samples represent two independent clonal cell lines obtained after *CTLA4*-KO. Exact *p*-values are shown for all indicated comparisons. The dash line corresponds to that threshold level (equals 1). (**d**) *CTLA4* activation in Jurkat^*CTLA4*+/+^, Jurkat^*CTLA4*+/−^, and Jurkat^*CTLA4*−/−^ cell lines following CRISPRa. The dash line corresponds to that threshold level (equals 1). All data are presented as box-and-whisker plots, with individual biological replicates shown as points. Sample size (n) for each group is indicated on the graph. Statistical analysis was performed as described in the Statistical Methods section. Levels of significance are indicated as **** *p* < 0.0001 and ns, not significant.

**Figure A4:**
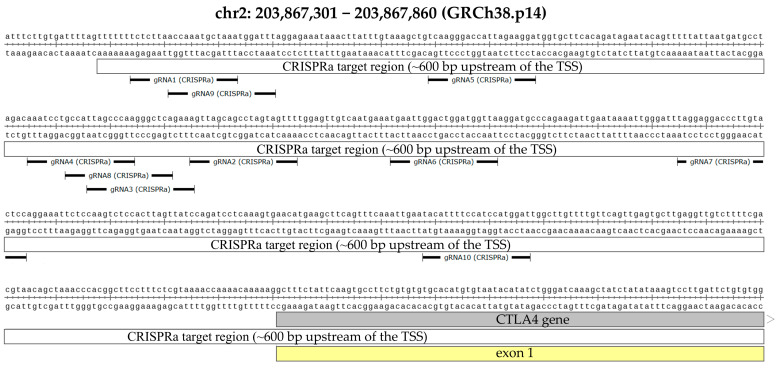
Schematic representation of the disposition of gRNAs used for CRISPRa. The white bar represents the *CTLA4* regulatory region; the grey bar represents the *CTLA4* gene body; the yellow bars represent the *CTLA4* gene exons; the pink bars represent the *CTLA4* coding region.

**Figure A5:**
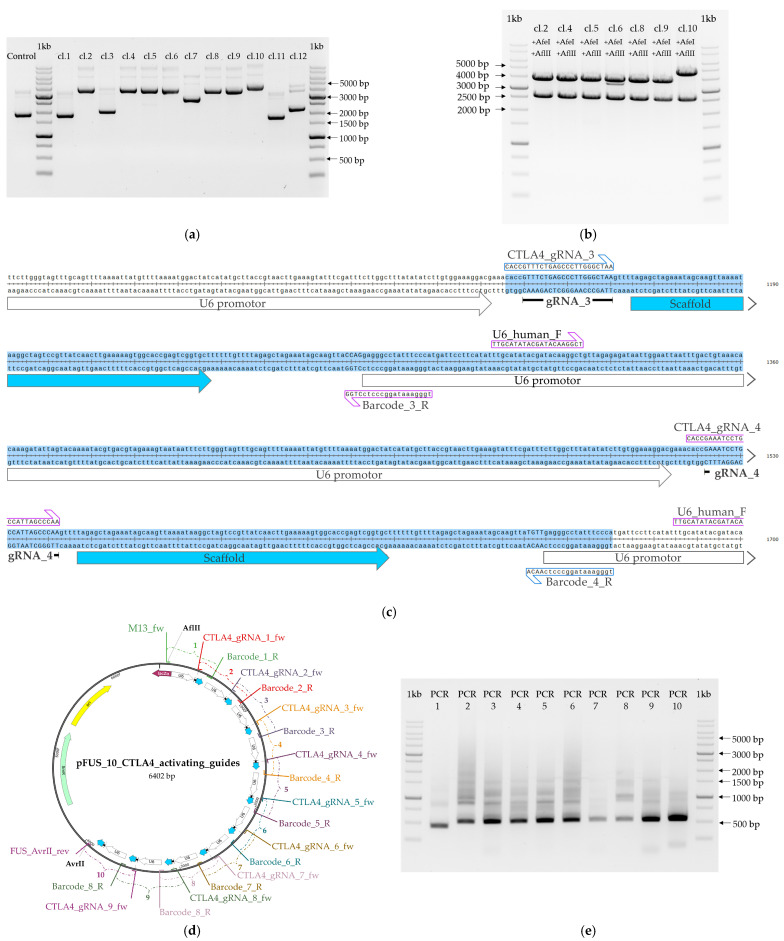

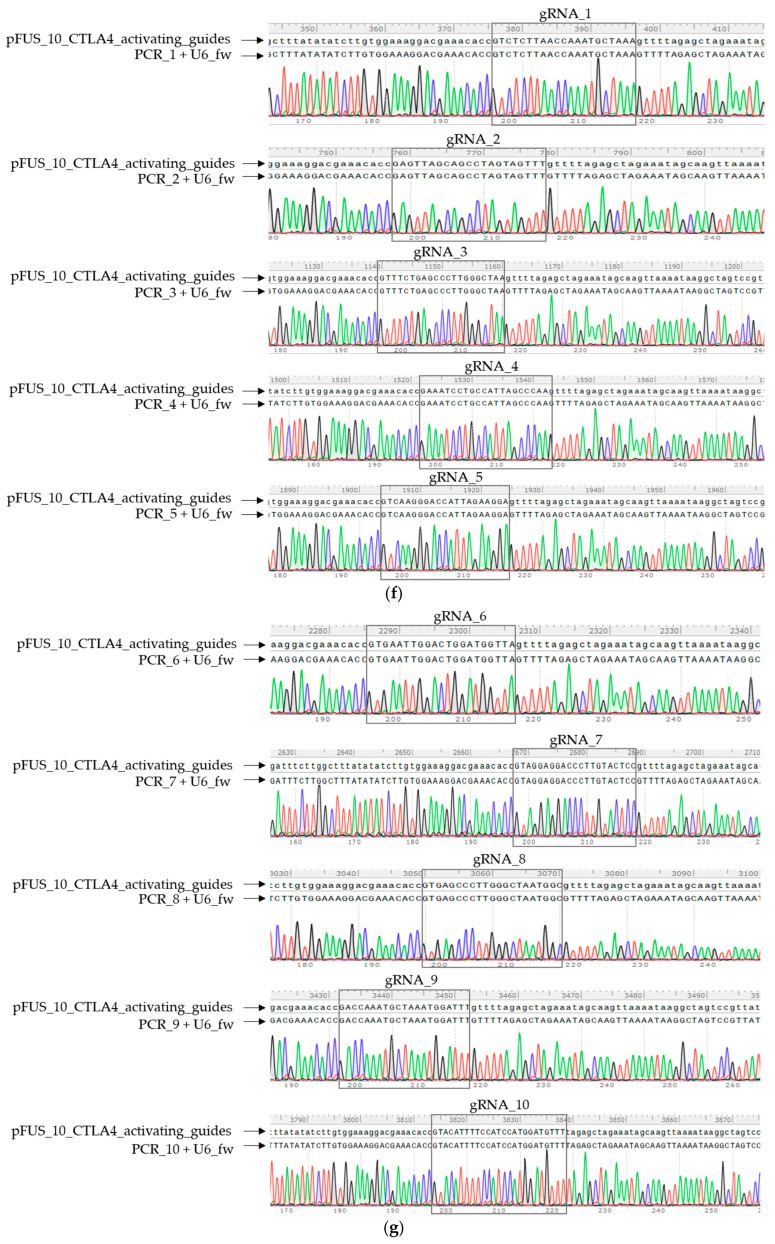
Stepwise structural validation of pFUS_10_CTLA4_activating_guides expression plasmid. (**a**) Screening of 12 bacterial colonies by agarose gel electrophoresis (supercoiled) with empty backbone control. (**b**) Analytical restriction digestion of selected clones, with AflII and AfeI showing the expected fragments (3872 bp and 2530 bp). (**c**,**d**) Schematic representation of the integrity PCR strategy used to verify individual gRNA expression cassettes, including primer design and Golden Gate barcode positioning. (**e**) Integrity PCR analysis of all ten gRNA cassettes. All integrity PCR reactions produced a dominant amplicon of the expected size. Minor non-specific amplification products were occasionally observed; however, gel purification of the expected bands, followed by Sanger sequencing, confirmed the correct guide sequences and U6-spacer-scaffold junctions for all cassettes. (**f**,**g**) Representative Sanger sequencing chromatograms of gel-purified PCR products. Colors correspond to nucleotide identity in Sanger sequencing chromatograms (A—green, T—red, C—blue, G—black).

**Figure A6:**
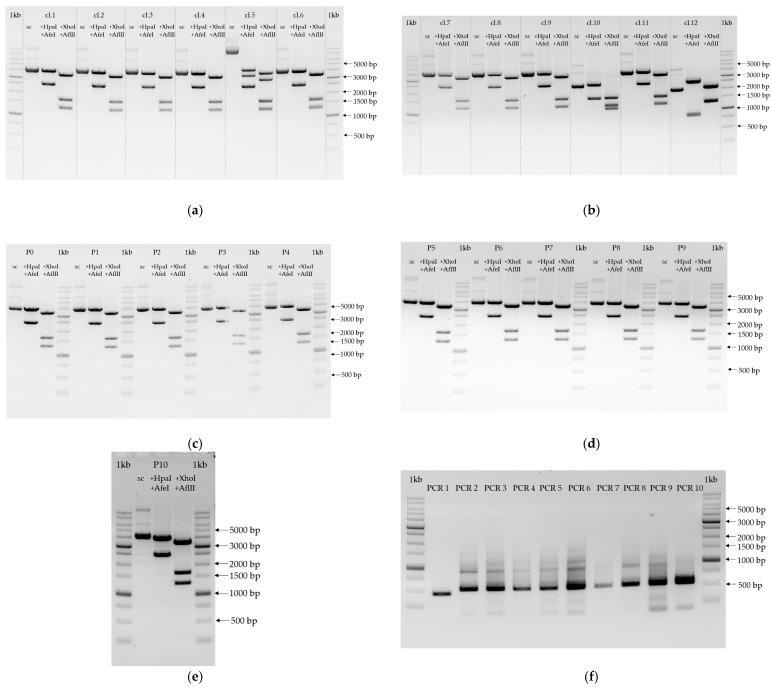

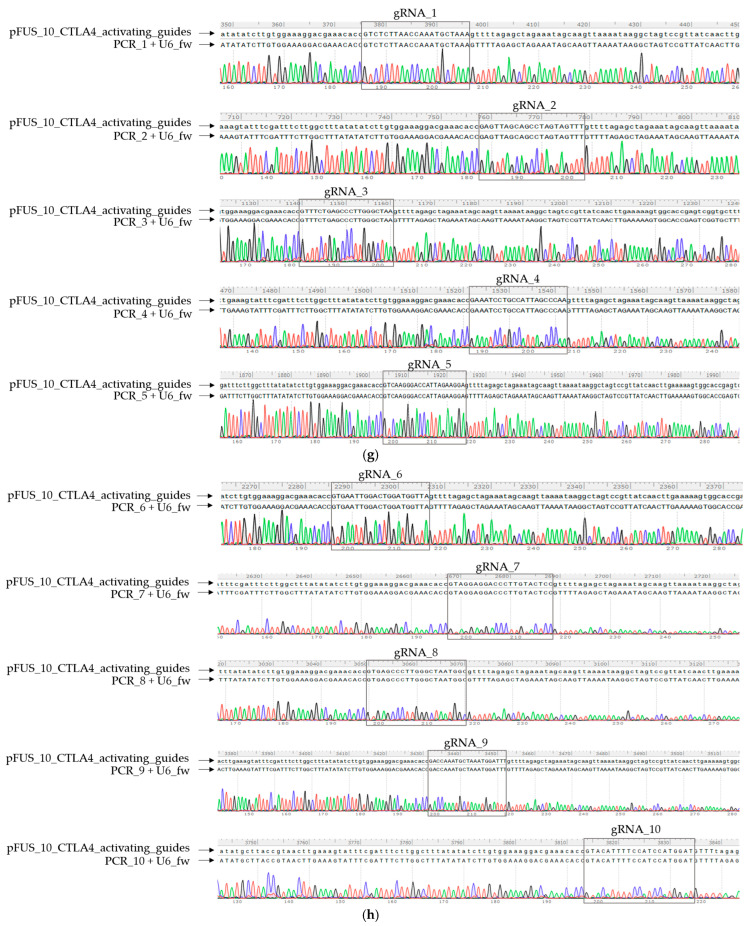
Structural stability analysis of the pFUS_10_CTLA4_activating_guides expression plasmid during bacterial propagation. (**a**,**b**) Screening of twelve independent colonies following re-transformation into *E. coli* DH5α cells. Native plasmid DNA agarose gel electrophoresis and analytical restriction digestions. (**c**–**e**) Stability assessment across ten consecutive overnight passages (P1–P10) under antibiotic selection; P0 used as a positive control. Native plasmid DNA agarose gel electrophoresis and two analytical restriction digestions: HpaI and AfeI (2477 bp and 3925 bp) and XhoI and AflII (1294 bp, 1630 bp, and 3478 bp). Nine of 12 colonies demonstrated the expected restriction pattern. (**f**) Integrity PCR analysis of all ten gRNA cassettes using plasmid DNA from passage 10 (P10). All reactions produced amplicons of the expected size. Minor non-specific products were occasionally observed; however, the expected bands were gel-purified and subjected to Sanger sequencing. (**g**,**h**) Representative Sanger sequencing chromatograms of gel-purified PCR products. Colors correspond to nucleotide identity in Sanger sequencing chromatograms (A—green, T—red, C—blue, G—black).

**Figure A7:**
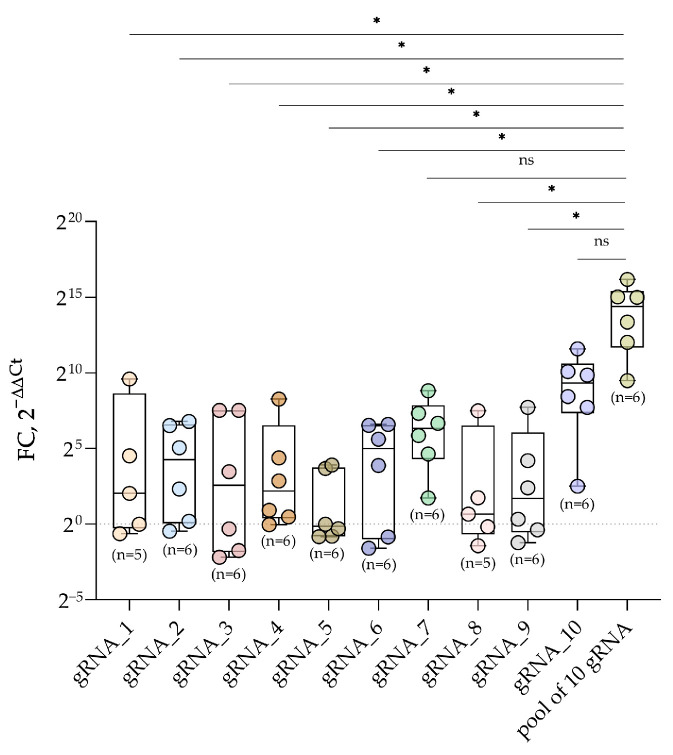
Comparison of individual gRNA-mediated *CTLA4* activation versus pooled gRNA strategy in HEK293T cells. *CTLA4* mRNA levels were quantified by RT-qPCR following CRISPRa using individual gRNAs (gRNA_1-gRNA_10) or a pool of 10 gRNAs. Expression values are shown as fold change relative to the control condition—intact HEK293T. Each dot represents an independent biological replicate. Individual gRNAs were compared to the pooled gRNA condition. Statistical analysis was performed as described in the Statistical Methods section. Levels of significance are indicated as * *p* < 0.05; ns, not significant.

In general terms, haploinsufficiency is a dominant condition, caused by insufficient expression of one remaining functional allele that fails to produce a wild-type phenotype. The deficiency of one allele expression might be a result of deletion, frame-shift, early stop, etc. The second allele in this case retained an intact ORF and, in theory, could be activated twofold to reach the expression levels of the wild-type phenotype. In the present study, we aimed to develop a strategy of epigenetic activation of *CTLA4* by CRISPRa as a possible treatment for *CTLA4* haploinsufficiency. The *CTLA4* gene consists of four exons and contains an untranslated region in the 5′end of the gene. We designed 10 gRNAs in the 5′-UTR of the *CTLA4* gene in positions between +300 bp and +500 bp upstream of the start codon (Figure 2a; Appendix A, Figure A4). The selected gRNAs were first cloned into separate pMA vectors, followed by assembling them into one vector by Golden Gate cloning. Correct assembly was confirmed by colony screening, analytical restriction digestion, integrity PCR, and Sanger sequencing (Appendix A, Table A2, Figure A5). The assembled vector was also assessed for the loss of repeating units—gRNA cassettes (Appendix A, Table A2, Figure A6). To address whether the use of multiple gRNAs was necessary, we compared the transcriptional activation efficiency of individual gRNAs to that of the pooled gRNA approach in HEK293T cells (Appendix A, Figure A7).

**Figure A8:**
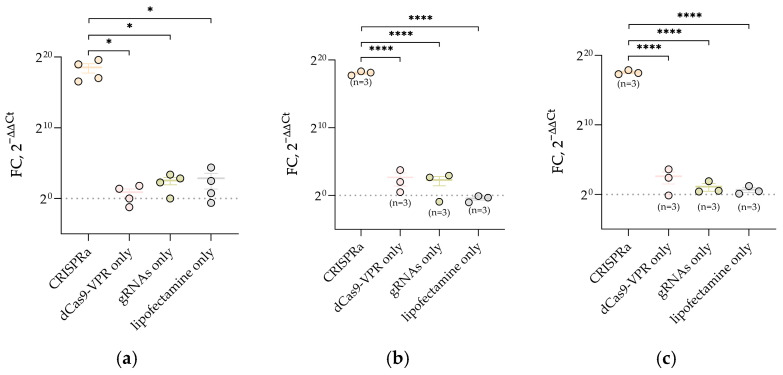

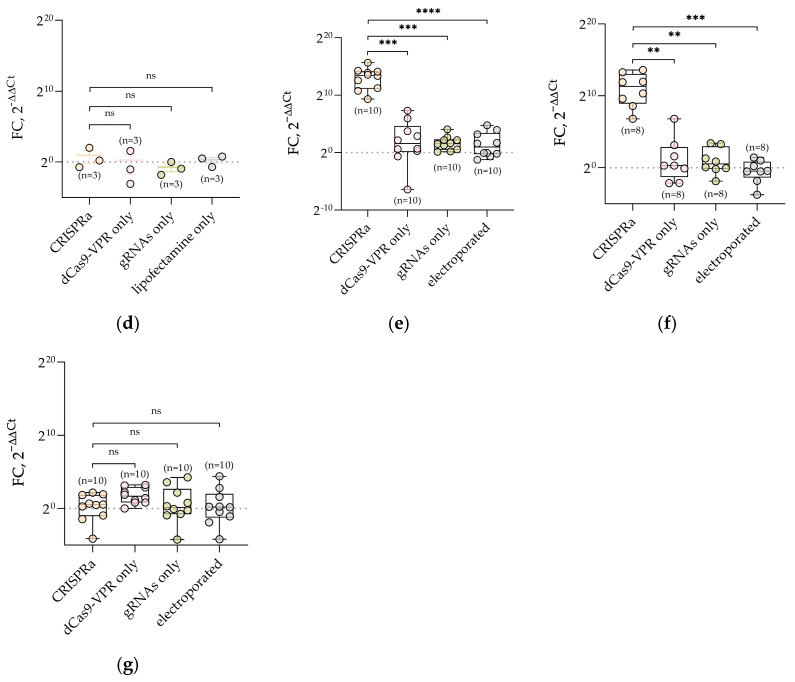
Graphical representation of CRISPRa controls in (**a**) HEK293T^*CTLA4*+/+^, (**b**,**c**) HEK293T^*CTLA4*+/−^, (**d**) HEK293T^*CTLA4*−/−^, (**e**) Jurkat^*CTLA4*+/+^, (**f**) Jurkat^*CTLA4*+/−^, and (**g**) Jurkat^*CTLA4*−/−^. HEK293T*^CTLA4^*^+/−^ samples represent two independent clonal cell lines obtained after *CTLA4*-KO. All data are presented as box-and-whisker plots, with individual biological replicates shown as points. Sample size (n) for each group is indicated on the graph. Statistical analysis was performed as described in the Statistical Methods section. Levels of significance are indicated as * *p* < 0.05, ** *p* < 0.01, *** *p* < 0.001, and **** *p* < 0.0001; ns, not significant.

For the CRISPRa protocol, we transfected HEK293T cells with two plasmids, one coding for activating gRNAs and the other coding for dCas9-VPR. In 72 h after transfection, we isolated the total RNA from the cells, followed by RT-qPCR. To estimate the upregulation rate of *CTLA4* expression, we used a primer pair located at the junction of exons 2 and 3 ending in the middle of exon 4 (Figure 2b). The CRISPRa protocol in both HEK293T*^CTLA4^*^+/+^ and HEK293T*^CTLA4^*^+/−^ resulted in a clear and reproducible increase in *CTLA4* mRNA levels (Figure 2c). The fold-change values formed compact clusters with low variability within each genotype group, indicating a stable activation response across biological replicates. Whilst the mean *CTLA4* expression in HEK293T*^CTLA4^*^+/+^ cells appeared slightly higher than in HEK293T*^CTLA4^*^+/−^, the difference did not reach statistical significance, confirming that a single functional allele is sufficient to support strong CRISPRa-induced *CTLA4* transcription. In contrast, HEK293T^*CTLA4*−/−^ cells showed no detectable *CTLA4* expression upon the CRISPRa protocol, with all replicates tightly clustered around the baseline (Figure 2c). These data demonstrate that the activation protocol is strictly dependent on the presence of a remaining coding allele and cannot induce expression if the entire *CTLA4* coding region is deleted. To verify the specificity of CRISPRa, we assessed the degree of *CTLA4* activation in the following control cells: untreated, gRNA-only treated, and dCas9-VPR-only treated. As expected, for HEK293T*^CTLA4^*^+/+^ and HEK293T*^CTLA4^*^+/−^, as well as HEK293T^*CTLA4*−/−^, the *CTLA4* activation was not detected in any of the controls (Appendix A, Figure A8a–d).

The pronounced elevation of *CTLA4* expression by CRISPRa in HEK293T confirmed its efficacy and allowed us to test the potency of the CRISPRa on Jurkat*^CTLA4^*^+/+^ cells as the closest model to T cells. To do so, we delivered CRISPRa vectors into Jurkat*^CTLA4^*^+/+^ by electroporation. After 72 h, we isolated the total RNA fraction and conducted RT-qPCR. As in the case of HEK293T*^CTLA4^*^+/+^, a similar pattern was observed in Jurkat*^CTLA4^*^+/+^ (Figure 2d). Both Jurkat*^CTLA4^*^+/+^ and Jurkat*^CTLA4^*^+/−^ cells responded to CRISPRa with a several-fold increase in *CTLA4* mRNA expression. Again, the variability within each group remained low, and the difference between Jurkat*^CTLA4^*^+/+^ and Jurkat*^CTLA4^*^+/−^ was not statistically significant. As expected, the Jurkat^*CTLA4*−/−^ cells did not show detectable induction. Also, similar to the CRISPRa protocol in HEK293T cell derivatives, we assessed *CTLA4* expression changes in the same control settings. Predictably, we detected no *CTLA4* expression changes in any of the controls (Appendix A, Figure A8e–g).

Together, these findings indicate that CRISPRa definitely activates transcription from the remaining *CTLA4* allele, regardless of whether cells are wild-type or the haploinsufficiency model. At the same time, cells lacking the *CTLA4* ORF remain unresponsive, providing an internal negative control of CRISPRa specificity.

To determine if *CTLA4* transcription activation is followed by *CTLA4* protein levels upregulation, we performed intracellular *CTLA4* staining 48 h after electroporation of CRISPRa constructs into Jurkat cells of three genotypes: Jurkat*^CTLA4^*^+/+^, Jurkat*^CTLA4^*^+/−^, and Jurkat^*CTLA4*−/−^ (Table 5). Intracellular staining was performed to assess total *CTLA4* protein levels, as *CTLA4* is predominantly located in intracellular vesicles. Both Jurkat*^CTLA4^*^+/+^ and Jurkat*^CTLA4^*^+/−^ responded to CRISPRa with an increase in the proportion of *CTLA4*^+^ cells, whereas Jurkat^*CTLA4*−/−^ remained at background levels (Table 5). Although the mean percentage of *CTLA4*^+^ cells was higher in Jurkat*^CTLA4^*^+/+^ compared to Jurkat*^CTLA4^*^+/−^, this difference did not reach statistical significance. Similarly, the difference between Jurkat*^CTLA4^*^+/−^ and Jurkat^*CTLA4*−/−^ did not reach statistical significance (Appendix A, Table A3). However, Jurkat*^CTLA4^*^+/−^ cells showed low but consistently detectable *CTLA4*^+^ fractions (2.5–7.8%), whereas Jurkat^*CTLA4*−/−^ remained at 0% (Table 5).

The difference between Jurkat*^CTLA4^*^+/+^ and Jurkat^*CTLA4*−/−^ reached statistical significance (Appendix A, Table A3), reflecting the presence versus absence of the coding sequence. Furthermore, a tendency toward increased *CTLA4* protein levels was observed in Jurkat*^CTLA4^*^+/−^ cells compared to Jurkat^*CTLA4*−/−^, although this did not reach statistical significance. The values follow the expected qualitative pattern and remain fully consistent with the CRISPRa-dependent activation observed in the transcript level analysis.

**Figure 3:**
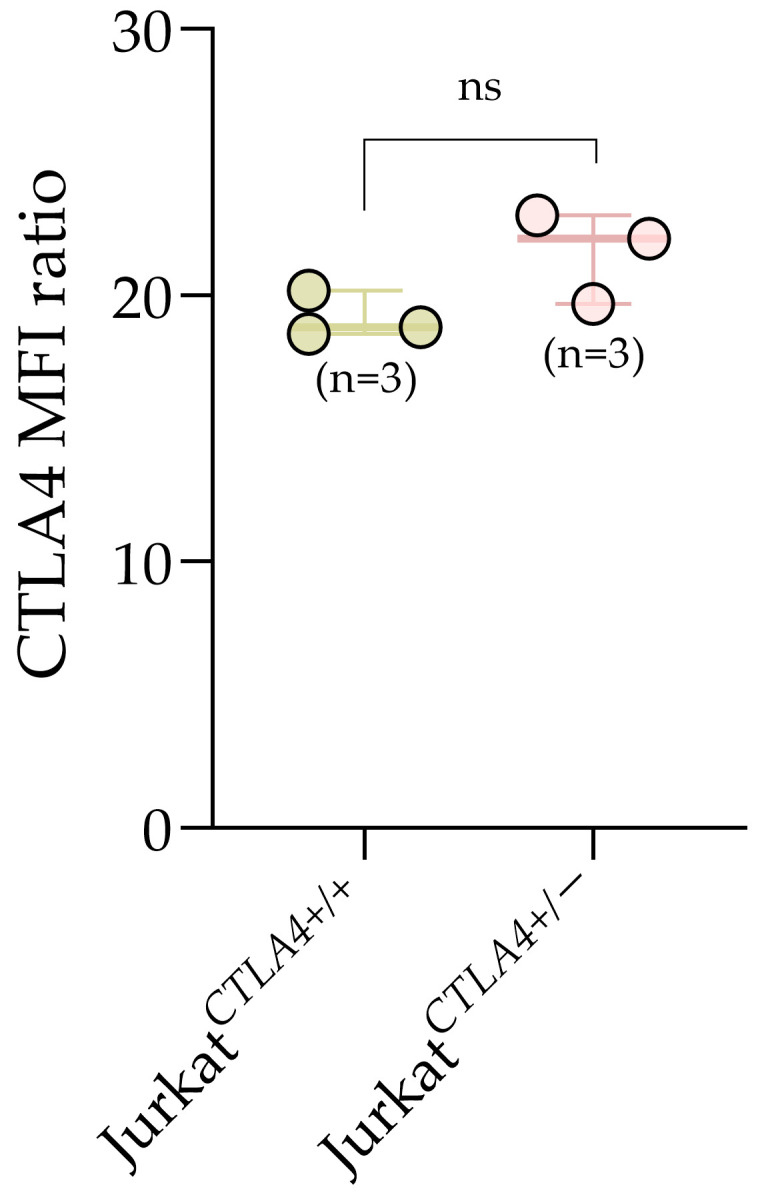
*CTLA4* protein levels quantified as the geometric mean fluorescence intensity ratio (geoMFI^+^/geoMFI^−^) upon CRISPRa. All data are presented as box-and-whisker plots, with individual biological replicates shown as points. Statistical analysis was performed as described in the Statistical Methods section. Levels of significance are indicated as ns, not significant.

To evaluate relative protein levels independently of background variation, we calculated the geometric MFI ratio (*CTLA4*^+^/*CTLA4*^−^) within each sample of genotypes: Jurkat*^CTLA4^*^+/+^ and Jurkat*^CTLA4^*^+/−^ (Figure 3). MFI ratio values were comparable between Jurkat*^CTLA4^*^+/+^ and Jurkat*^CTLA4^*^+/−^, with a slight but non-significant tendency toward a higher value. This suggests that activation of a single remaining allele yields sufficient transcript output to produce detectable intracellular protein at the 48 h time point.

### 3.3. Targeted CTLA4 Activation by CRISPRa Compared to Non-Specific Stimulation in Jurkat^CTLA4+/+^

To assess the specificity and physiological validity of *CTLA4* activation, we compared *CTLA4* protein upregulation upon CRISPRa to the response to non-specific stimulation using PMA/ionomycin in Jurkat*^CTLA4^*^+/+^ cells. Protein expression levels were evaluated at four time points: 16–18 h, 24 h, 48 h, and 72 h upon either stimulation or CRISPRa.

To assess cellular activation status, the general early activation marker CD69 was measured by surface staining, whereas *CTLA4* protein levels were quantified by intracellular staining after fixation and permeabilization.

**Figure 4:**
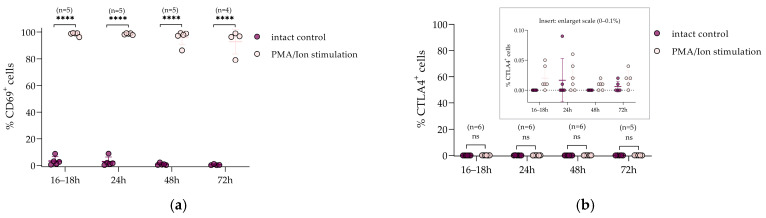

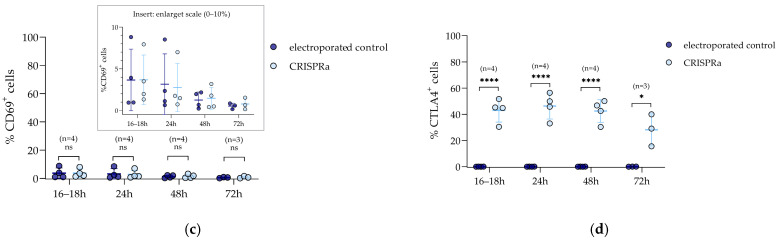
Dynamics of *CTLA4* and CD69 expression in the Jurkat cell line upon stimulation. (**a**). Percentage of CD69^+^ cells after PMA/Ionomycin stimulation compared with intact control at four time points (16–18 h, 24 h, 48 h, and 72 h). (**b**) Percentage of *CTLA4*^+^ cells under the same conditions; insert shows an enlarged 0–0.1% scale. (**c**) Percentage of CD69^+^ cells upon CRISPRa activation compared with electroporated control; insert shows an enlarged 0–10% scale. (**d**) Percentage of *CTLA4*^+^ cells upon CRISPRa activation compared with electroporated control. Dark symbols—control groups (intact or electroporated); light symbols—stimulated groups (PMA/Ionomycin or CRISPRa). Data are presented as individual biological replicates with mean ± SD. Sample size (n) for each group is indicated on the graph. Statistical analysis was performed as described in the Section 2. Levels of significance are indicated as * *p* < 0.05, and **** *p* < 0.0001; ns, not significant.

PMA/ionomycin stimulation resulted in significant cell activation: the CD69^+^ cell proportion exceeded 90% (Figure 4a), but *CTLA4* protein expression was not detected at any of the measured time points (Figure 4b). In contrast, CRISPRa-mediated activation resulted in low CD69^+^ cell proportions (<10%) (Figure 4c), while intracellular staining revealed significant *CTLA4* induction (30–50%) (Figure 4d). *CTLA4*^+^ cells appeared as early as 16–18 h, peaked at 24 h, remained elevated at 48 h, and declined slightly by 72 h. Importantly, the magnitude of induction and the close clustering of biological replicates highlight the representativeness of the activation directed at the *CTLA4* promoter across independent experiments.

Together, these results confirm that CRISPRa drives specific and efficient activation of the endogenous *CTLA4* promoter, in contrast to non-specific stimulation in Jurkat cells. Moreover, the CRISPRa protocol enables restoration of *CTLA4* expression, even in contexts where non-specific stimulation methods prove insufficient.

### 3.4. Relative Expression of CTLA4

**Figure 5:**
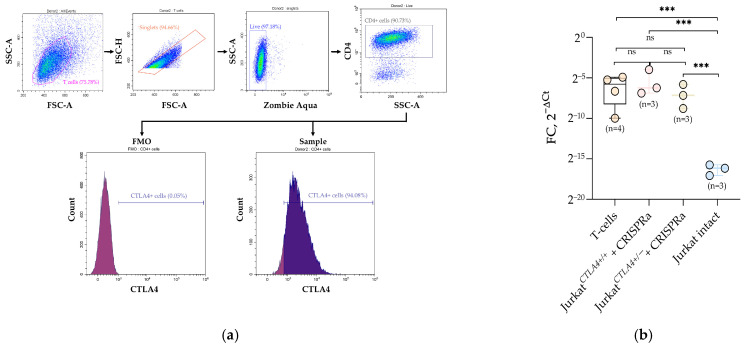
Relative *CTLA4* expression in T cells and Jurkat cell lines upon CRISPRa activation. (**a**) Representative gating example of MACS separated CD4^+^CD25^−^ T cells on day 10 of cultivation. Total T cell subset was gated for single cells, live cells (Zombie Aqua^−^), and non-CD4^+^ cells. *CTLA4* expression was analyzed in the CD4^+^ cell gate. FMO control was used to define the *CTLA4*^+^ gate. (**b**) *CTLA4* mRNA levels (2^−∆Ct^) measured by RT-qPCR in activated primary T cells, Jurkat*^CTLA4^*^+/+^, and Jurkat*^CTLA4^*^+/−^ upon CRISPRa and untreated intact Jurkat cells (baseline control). Expression levels were normalized to *RPL32* and *GAPDH* and represent normalized expression within each group. No additional calibrator sample was applied, as no shared biological baseline existed across all analyzed groups. Data are presented as individual biological replicates with mean ± SD. Sample size (n) for each group is indicated on the graph. Statistical analysis was performed as described in the Statistical Methods section. Levels of significance are indicated as *** *p* < 0.001; ns, not significant.

Following the demonstration that the CRISPRa protocol reliably and specifically activates transcription from the remaining haplo *CTLA4* allele to levels statistically indistinguishable between wild-type and haploinsufficiency models, we aimed to determine whether these expression levels fall within the physiological range. For this, we isolated CD3^+^ T cells from PBMCs and subjected them to a standard activation protocol known to induce stable endogenous *CTLA4* expression. The purpose of this experiment was to obtain primary T cells that were naturally activated and would serve as a physiological reference. Intracellular staining and flow cytometry confirmed the presence of a distinct *CTLA4*^+^ population among activated primary T cells (Figure 5a).

We performed RT-qPCR on four groups: activated primary T cells, CRISPRa-treated Jurkat*^CTLA4^*^+/+^, CRISPRa-treated Jurkat*^CTLA4^*^+/−^, and untreated intact Jurkat*^CTLA4^*^+/+^ cells (baseline control) (Figure 5b). For intergroup comparison, normalized 2^−∆Ct^ values were statistically analyzed without applying a single reference calibrator condition, as activated primary T cells represent a distinct induced transcriptional state. *CTLA4* mRNA levels in both Jurkat*^CTLA4^*^+/+^ as well as Jurkat*^CTLA4^*^+/−^ cells upon CRISPRa were comparable to activated primary T cells; moreover, no statistically significant differences were detected among these three assessed groups. Thus, CRISPRa-induced activation in Jurkat cells does not lead to overexpression but instead reaches the range characteristic of physiological *CTLA4* induction in T cells.

Taken together, these results demonstrate that our CRISPRa protocol not only restores *CTLA4* expression in the haploinsufficiency model but also elevates it to a physiologically relevant level comparable to that of naturally activated T cells, highlighting the therapeutic significance of this approach.

### 3.5. Off-Target Effects of CTLA4 Epigenetic Activation

**Figure 6:**
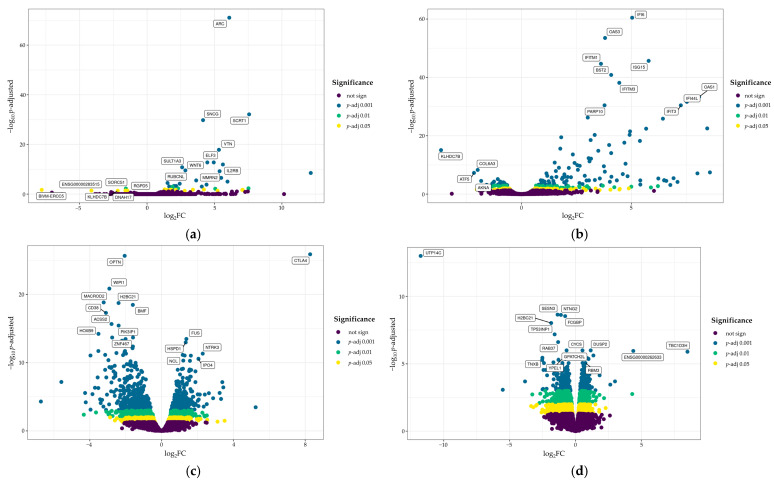
Off-target effects of the CRISPRa protocol. Volcano plots demonstrating upregulated and downregulated genes upon *CTLA4* CRISPRa (vs. intact control) in HEK293T*^CTLA4^*^+/+^ (**a**), HEK293T*^CTLA4^*^−/−^ (**b**), Jurklat*^CTLA4^*^+/+^ (**c**), and Jurkat*^CTLA4^*^−/−^ (**d**).

**Figure A9:**
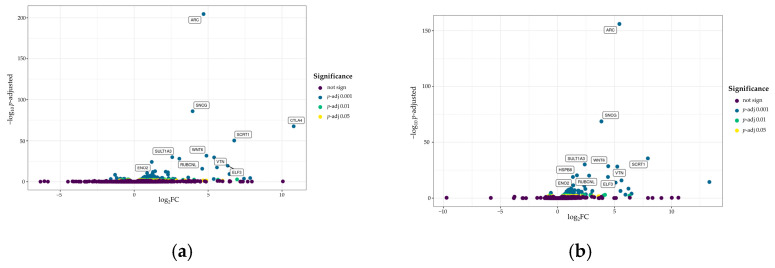

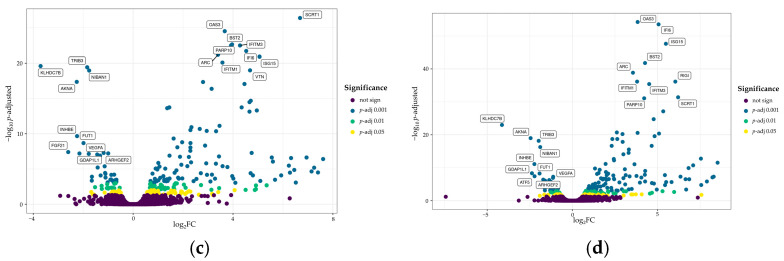
CRISPRa off-target controls in HEK293T*^CTLA4^*^+/+^ and HEK293T^*CTLA4*−/−^. (**a**) HEK293T*^CTLA4^*^+/+^: CRISPRa vs. gRNA-only control; (**b**) HEK293T*^CTLA4^*^+/+^: CRISPRa vs. dCas9-VPR-only control; (**c**) HEK293T^*CTLA4*−/−^: CRISPRa vs. gRNA-only control; (**d**) HEK293T^*CTLA4*−/−^: CRISPRa vs. dCas9-VPR-only control.

To address the issue of possible off-target effects, we conducted whole transcriptome analysis upon *CTLA4* CRISPRa. Initially, to assess the impact of both the CRISPRa protocol as well as *CTLA4* expression itself, we evaluated gene expression changes in HEK293T^*CTLA4*+/+^ cells upon *CTLA4* CRISPRa. For this, we assessed differential expression by comparing the CRISPRa with the following controls: untreated, gRNA-only, and dCas9-VPR-only. First, CRISPRa vs. untreated control pair comparisons revealed 38 upregulated and 6 downregulated genes with padj < 0.05 (Figure 6a). Further comparison of CRISPRa with gRNA-only control gave 131 upregulated and 30 downregulated genes with padj < 0.05 (Appendix A, Figure A9a). Finally, comparison of CRISPRa and dCas9-VPR-only control identified 112 upregulated and 6 downregulated genes with padj < 0.05 (Appendix A, Figure A9b).

Next, to assess the impact of CRISPRa itself on gene expression, we performed whole transcriptome analysis in HEK293T^*CTLA4*−/−^ cells. As in HEK293T^*CTLA4*+/+^, we compared CRISPRa with the three above-mentioned controls. In this case, in the “CRISPRa—untreated control” pair, we observed 217 upregulated and 127 downregulated genes with padj < 0.05 (Figure 6b). Next, comparison of CRISPRa with gRNA-only control showed 185 upregulated and 74 downregulated genes with padj < 0.05 (Appendix A, Figure A9c). Finally, the comparison between CRISPRa and dCas9-VPR control showed 196 upregulated and 78 downregulated genes with padj < 0.05 (Appendix A, Figure A9d).

**Figure A10:**
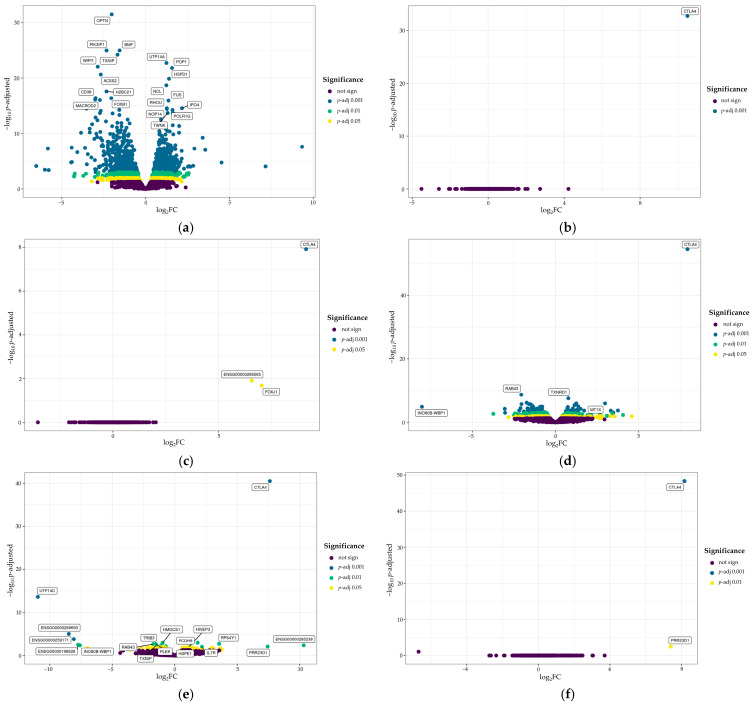

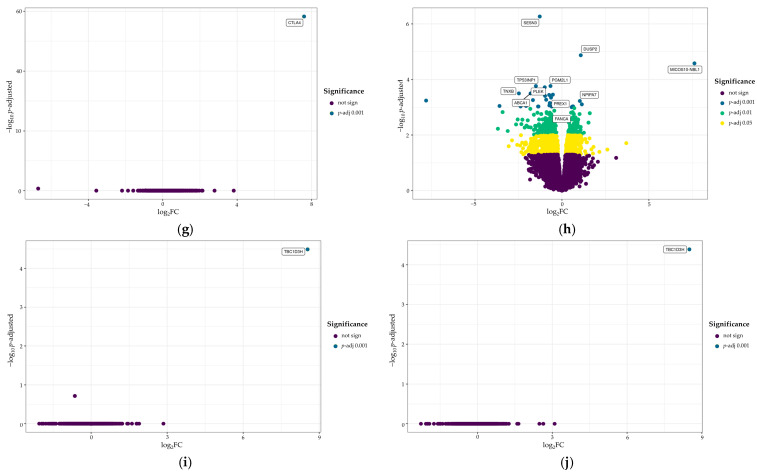
Off-target effects in Jurkat*^CTLA4^*^+/+^ and Jurkat^*CTLA4*−/−^ upon CRISPRa. (**a**) Jurkat*^CTLA4^*^+/+^: CRISPRa vs. electroporation control; (**b**) Jurkat*^CTLA4^*^+/+^: CRISPRa vs. gRNA-only control; (**c**) Jurkat*^CTLA4^*^+/+^: CRISPRa vs. dCas9-VPR-only control; (**d**) Jurkat*^CTLA4^*^+/+^: monoclonal derivative CRISPRa vs. untreated control; (**e**) Jurkat*^CTLA4^*^+/+^: monoclonal derivative CRISPRa vs. electroporated control; (**f**) Jurkat*^CTLA4^*^+/+^: monoclonal derivative CRISPRa vs. gRNA-only control; (**g**) Jurkat*^CTLA4^*^+/+^: monoclonal derivative CRISPRa vs. dCas9-VPR control; (**h**) Jurkat^*CTLA4*−/−^: CRISPRa vs. electroporated control; (**i**) Jurkat^*CTLA4*−/−^: CRISPRa vs. gRNA-only control; (**j**) Jurkat^*CTLA4*−/−^: CRISPRa vs. dCas9-VPR control.

After this, we addressed the same question in Jurkat*^CTLA4^*^+/+^ cells. In this experiment, a control in which cells were electroporated without plasmid DNA introduction was added to the controls described above. First, the comparison in “CRISPRa vs. untreated control” pair demonstrated 934 upregulated and 938 downregulated genes with padj < 0.05 (Figure 6c). Next, in the “CRISPRa vs. electroporated control” pair, we observed 1296 upregulated and 1238 downregulated genes with padj < 0.05 (Appendix A, Figure A10a). Surprisingly, comparisons in pairs “CRISPRa vs. gRNA-only control” and “CRISPRa vs. dCas9-VPR-only control” resulted in only *CTLA4* activation and either 0 or 2 differentially expressed off-target genes, respectively (Appendix A, Figure A10b,c). To independently verify this phenomenon, we generated a monoclonal wild-type Jurkat derivative and conducted CRISPRa with the same control settings. In this case, we observed 1159 upregulated and 646 downregulated genes with padj < 0.05 in the pair “CRISPRa vs. untreated control” (Appendix A, Figure A10d), as well as 52 upregulated and 79 downregulated genes with padj < 0.05 in the pair “CRISPRa vs. electroporated control” (Appendix A, Figure A10e). Again, the comparison in pairs “CRISPRa vs. gRNA-only control” and “CRISPRa vs. dCas9-VPR” demonstrated only *CTLA4* activation and, in the case of “CRISPRa vs. gRNA-only control”, 1 upregulated off-target gene, namely *PRR23D1* (Appendix A, Figure A10f,g).

Finally, we aimed to assess transcriptome-wide off-target effects in Jurkat^*CTLA4*−/−^ cells. In the pair “CRISPRa vs. untreated control”, we observed 1425 upregulated and 1321 downregulated genes with padj < 0.05 (Figure 6d). Next, in the comparison of the “CRISPRa vs. electropotared control” pair, we identified 1066 upregulated and 1086 downregulated genes with padj < 0.05 (Appendix A, Figure A10h). Similarly with all previous Jurkat derivatives, comparison in both pairs “CRISPRa vs. gRNA-only control” and “CRISPRa vs. dCas9-VPR control” gave just 1 upregulated off-target gene, different from that in Jurkat*^CTLA4^*^+/+^, namely *TBC1D3H* (Appendix A, Figure A10i,j).

To better characterize these transcriptome changes, we performed gene set enrichment analysis (GSEA) using the KEGG and Hallmark datasets. GSEA was performed on gene lists from the comparison of CRISPRa cells versus intact cells, separately for Jurkat*^CTLA4^*^+/+^ and Jurkat^*CTLA4*−/−^.

**Figure 7:**
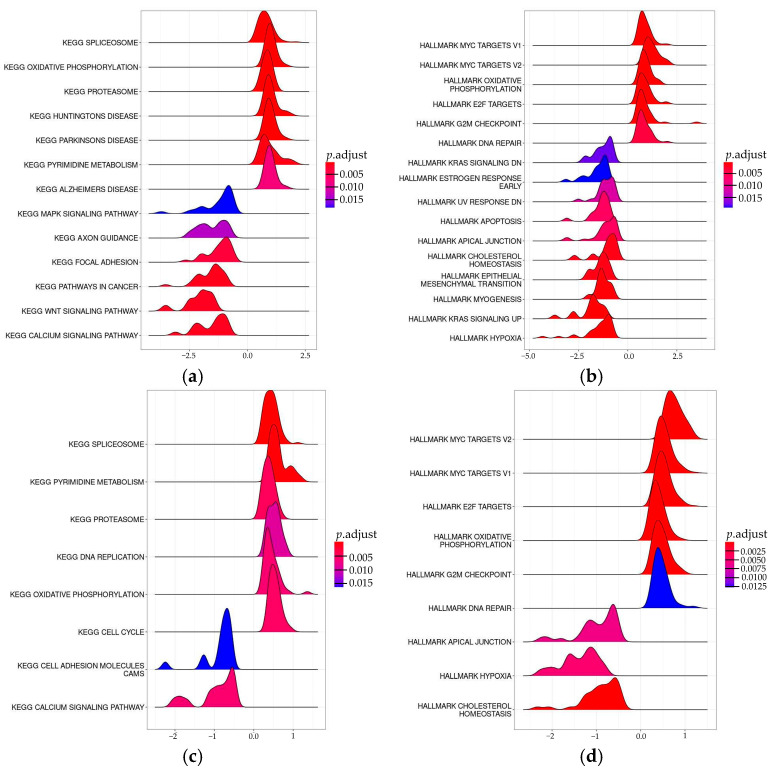
KEGG and Hallmark pathway enrichment analysis (GSEA) of transcriptomic changes induced by the CRISPRa protocol (adjusted *p*-values, *p*-adj). (**a**) KEGG enrichment in Jurkat*^CTLA4^*^+/+^ (CRISPRa vs. intact). (**b**) Hallmark enrichment in Jurkat*^CTLA4^*^+/+^ (CRISPRa vs. intact). (**c**) KEGG enrichment in Jurkat^*CTLA4*−/−^ (CRISPRa vs. intact). (**d**) Hallmark enrichment in Jurkat^*CTLA4*−/−^ (CRISPRa vs. intact).

In both genotypes, KEGG pathway analysis revealed statistically significant enrichment of basic cellular pathways such as the spliceosome, pyrimidine metabolism, proteasome, and oxidative phosphorylation pathways, accompanied by downregulation of calcium signaling (Figure 7a,c). These shared pathway changes likely reflect a shared transcriptional response to CRISPRa-mediated gene activation.

In addition, differences between genotypes were observed. In Jurkat*^CTLA4^*^+/+^ cells, gene sets associated with neurodegenerative pathways were enriched, whereas cancer-related pathways, as well as WNT signaling, were specifically downregulated. In contrast, these effects were not observed in Jurkat^*CTLA4*−/−^ cells, which may reflect differences in transcriptional changes following CRISPRa, depending on the *CTLA4* allele.

Hallmark pathway analysis further supported this conclusion, revealing enrichment of MYC targets (v1 and v2), E2F targets, oxidative phosphorylation, G2M checkpoint pathways, together with downregulation of apical junction, hypoxia, and cholesterol homeostasis in both genotypes (Figure 7b,d). Notably, DNA repair pathways were enriched, while apoptosis, epithelial–mesenchymal transition, myogenesis, and KRAS signaling were specifically downregulated in Jurkat*^CTLA4^*^+/+^ cells.

Taken together, these results suggest that the observed transcriptomic changes likely reflect both general CRISPRa-associated activation, genotype-dependent downstream effects related to *CTLA4* genetic background, and non-specific off-target activity.

## 4. Discussion

In this paper, we examined CRISPRa as a potential therapeutic strategy for *CTLA4* haploinsufficiency in cell models. The developed CRISPRa approach uses dCas9-VPR to activate an intact coding *CTLA4* allele in the *CTLA4*^+/−^ genotype. Across all experiments, our data consistently demonstrated that the CRISPRa protocol induces a robust and highly reproducible increase in *CTLA4* transcription, independent of the specific genetic background. Notably, comparison of each individual gRNA with a pool of 10 gRNAs demonstrated the most consistent and reproducible *CTLA4* transcriptional activation within a physiologically relevant range. Indeed, in activation experiments using each gRNA separately, only a few gRNAs were capable of *CTLA4* expression activation (Appendix A, Figure A7). This supports the utility of the pooled strategy in minimizing variability among individual guides. Interestingly, the gRNA showing the strongest individual activation effect overlapped with an annotated NFAT binding site, a known transcriptional regulator of *CTLA4*.

Both HEK293T and Jurkat *CTLA4* haploinsufficiency models responded to CRISPRa at levels comparable to corresponding wild-type controls, confirming that a single intact coding allele may be sufficient to support physiologically relevant *CTLA4* expression upon epigenetic activation. Since *CTLA4* deficiency can result from a variety of genotypes, it is crucial to highlight that the CRISPRa is only suitable for cases of true haploinsufficiency. This is an important condition for including/excluding a patient in plausible therapy, since if a dominant-negative variant of *CTLA4* is present, it will also be activated, leading to adverse effects. Thus, in the future, when using epigenetic therapy approaches, thorough patient genotyping is necessary.

Notably, our data also indicate that CRISPRa-mediated activation can operate even under conditions in which pharmacological stimulation fails to induce *CTLA4* expression. Jurkat*^CTLA4^*^+/+^ cells did not upregulate *CTLA4* upon PMA/ionomycin stimulation, consistent with their limited ability to demonstrate a complete activation response under such non-specific stimulation conditions [38,39]. Jurkat cells are known to carry multiple genomic mutations, affecting TCR signaling, genome stability, and O-linked glycosylation, and a heterozygous stop mutation in the *CTLA4* coding sequence has been described [40], which may contribute to impaired physiological *CTLA4* protein expression. These molecular features may partially account for the limited *CTLA4* upregulation observed upon PMA/ionomycin stimulation.

In contrast, the same cells responded robustly to CRISPRa. This observation suggests that epigenetic activation can drive *CTLA4* transcription independently of classical activation pathways, at least in cases where PMA/ionomycin stimulation is insufficient. Also, it is critical that the *CTLA4* expression levels upon CRISPRa treatment do not exceed *CTLA4* physiological expression in T cells. This is especially important, since the excess of *CTLA4* protein can disrupt the balance of the immune response and reduce the therapy efficiency, as well as lead to different autoimmune diseases. Maintaining *CTLA4* expression levels within a normal range ensures optimal T cells function, supporting their role in maintaining immune homeostasis and preventing undesirable side effects. To determine the physiological relevance of CRISPRa-driven expression levels, we compared CRISPRa-treated Jurkat cells to activated primary human T cells. The similarity in *CTLA4* mRNA levels across these groups indicates that CRISPRa does not cause overexpression but elevates *CTLA4* to a range characteristic of natural T-cell activation. This finding is particularly important for potential therapeutic applications, since achieving physiologically appropriate expression is often more desirable than strong overexpression.

Another important issue of epigenetic editing by CRISPRa is ensuring that the gene transcription activation leads to an increase in protein abundance. In the current study, we performed intracellular *CTLA4* staining to assess *CTLA4* protein expression from the activated *CTLA4* allele. Indeed, we showed that CRISPRa leads to an increase in the intracellular abundance of *CTLA4* protein. However, this measurement alone does not directly demonstrate *CTLA4* activity. Thus, the limitation of the present study is that it focuses on the targeted increasing of *CTLA4* transcription and protein abundance rather than direct assessment of its immune checkpoint function. Further functional studies will be required to confirm that the induced *CTLA4* performs its expected physiological roles.

**Figure A11:**
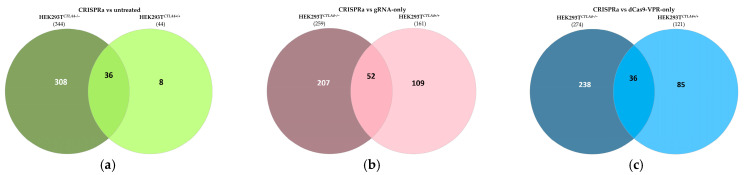
Off-target gene sets in HEK293T^*CTLA4*−/−^ and HEK293T*^CTLA4^*^+/+^. Venn diagrams demonstrating off-target gene sets upon CRISPRa compared with (**a**) untreated, (**b**) gRNA-only, and (**c**) dCas9-VRP-only controls.

**Figure A12:**
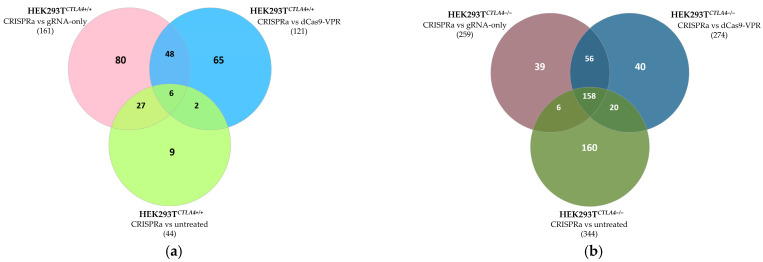
Off-target gene sets in HEK293T^*CTLA4*−/−^ and HEK293T*^CTLA4^*^+/+^. Venn diagrams showing overlaps between off-target gene sets in CRISPRa compared to different controls in (**a**) HEK293T*^CTLA4^*^+/+^and (**b**) HEK293T^*CTLA4*−/−^.

The next issue with CRISPRa is the problem of off-target effects. In CRISPR-based therapy, gRNAs can potentially target effector proteins to various genomic positions that differ from desired loci by one or several mismatches. In the case of gene editing with DNA breaks, these off-targets can lead to undesirable mutations in functionally important genomic loci. Although CRISPRa uses a catalytically inactive dCas9 protein, when targeted to off-target loci, it can alter gene expression and lead to deleterious effects. To prevent these problems, each CRISPR-based protocol needs to be tested for potential off-targets. In the current study, we performed whole transcriptome analysis following CRISPRa-mediated activation of the *CTLA4* gene. To differentiate off-target effects of CRISPRa from the effects of *CTLA4* activation itself on gene expression, we examined transcriptomic changes in HEK293T*^CTLA4^*^+/+^ and HEK293T^*CTLA4*−/−^ upon CRISPRa. When comparing the “CRISPRa vs. untreated” pairs between the two genotypes, we observed that for HEK293T*^CTLA4^*^+/+^, 36 of the 44 genes overlap with the DE gene set from HEK293T^*CTLA4*−/−^ (Appendix A, Figure A11 and Figure A12). This observation may indicate that *CTLA4* expression itself makes only a minor contribution to CRISPRa-driven transcriptomic changes. Next, we compared “CRISPRa vs. gRNA-only” off-target gene sets between HEK293T*^CTLA4^*^+/+^ and HEK293T^*CTLA4*−/−^, and the comparison resulted in partial gene set overlaps. The same pattern was also observed for the comparison of “CRISPRa vs. dCas9-VPR-only” off-target gene sets (Appendix A, Figure A11b,c). Moreover, gene set comparison between “CRISPRa vs. untreated” and both “CRISPRa vs. gRNA-only” and “CRISPRa vs. dCas9-VPR only” in both genotypes also gave overlaps (Appendix A, Figure A12). Thus, we demonstrated that in the case of HEK293T derivatives, the CRISPRa induces overlapping transcriptomic changes.

**Figure A13:**
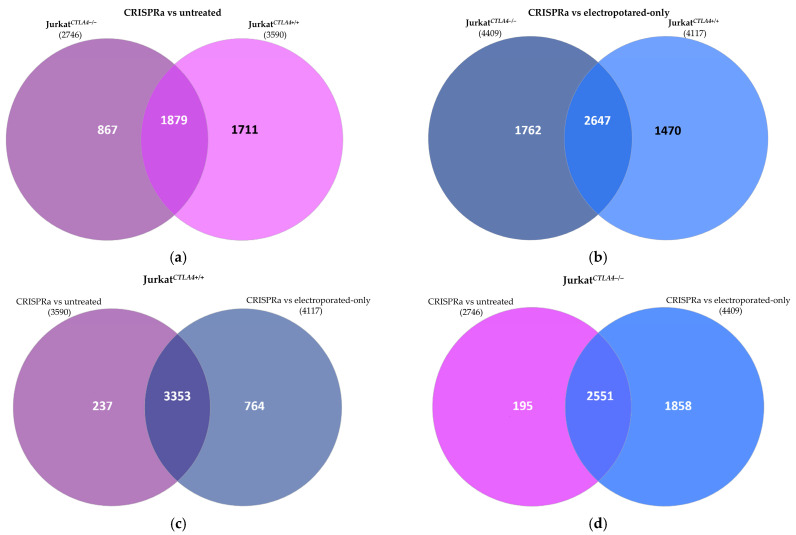
Off-target genes for Jurkat*^CTLA4^*^+/+^ and Jurkat^*CTLA4*−/−^. Venn diagrams demonstrating overlaps between comparisons in pairs (**a**) “CRISPRa vs. untreated”, (**b**) “CRISPRa vs. electroporated-only”, (**c**) “CRISPRa vs. gRNA-only”, and (**d**) “CRISPRa vs. dCas9-VPR-only”.

**Figure A14:**
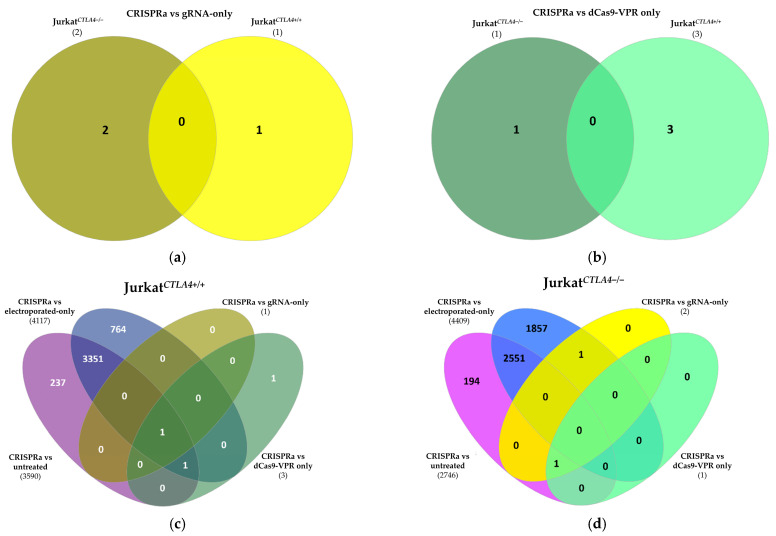
Off-target transcriptomic changes in Jurkat*^CTLA4^*^+/+^ and Jurkat^*CTLA4*−/−^. Venn diagrams demonstrating overlapping off-target gene sets in pairs “CRISPRa vs. untreated” and “CRISPRa vs. electroporated-only” in Jurkat*^CTLA4^*^+/+^ (**a**) and Jurkat^*CTLA4*−/−^ (**b**). Venn diagrams demonstrating overlaps of off-target genes in CRISPRa compared to all controls in Jurkat*^CTLA4^*^+/+^ (**c**) and Jurkat^*CTLA4*−/−^ (**d**).

To assess possible off-target effects in Jurkat cell line derivatives, we performed the same set of comparisons in Jurkat*^CTLA4^*^+/+^ and Jurkat^*CTLA4*−/−^. In this case, comparing the “CRISPRa vs. untreated” pair with the “CRISPRa vs. electroporated-only” pair resulted in significant overlaps both between the two genotypes and within each genotype (Appendix A, Figure A13). Interestingly, we found almost no differences when comparing “CRISPRa vs. gRNA-only” and “CRISPRa vs. dCas9-VPR-only” within both genotypes (Appendix A, Figure A14a,b). Moreover, when all four CRISPRa-control comparisons were evaluated, a considerable number of off-target genes were observed only in “CRISPRa vs. untreated” and “CRISPRa vs. electroporated-only” cases (Appendix A, Figure A14c,d). Since the observed pattern is repeated in Jurkat cells of both genotypes, it can be assumed that the introduction of plasmid DNA into cells itself leads to transcriptome changes. Thus, CRISPRa itself appears to induce limited off-target transcriptomic effects in Jurkat cells under the conditions tested.

In addition, we performed pathway enrichment analysis to better characterize transcriptomic changes observed after CRISPRa. Gene enrichment analysis revealed alterations in multiple pathways in both Jurkat*^CTLA4^*^+/+^ and Jurkat^*CTLA4*−/−^ cells when CRISPRa cells were compared to intact controls. While several enriched pathways were shared between genotypes, specific genotypic differences were also observed, suggesting that the CRISPRa transcriptional response may depend on *CTLA4* genetic background.

Nevertheless, potential off-target effects remain an important limitation of CRISPR-based systems and may arise from imperfect gRNA binding to partially homologous genomic regions. To minimize such effects, careful design of gRNAs, the use of computational prediction tools, and improved delivery methods are widely applied [41,42]. In addition, efficient and targeted delivery of CRISPR components plays a critical role in reducing unintended genomic interactions.

When discussing CRISPR-based studies, careful consideration must be given to developing an effective genetic vector system. Several delivery systems have been developed for CRISPR-based platforms, including viral vectors, transposon-based systems, electroporation, nanocarriers, hydrogels, and microfluidics-based approaches [43,44]. Hydrogels, a recently emerging delivery system, have already demonstrated their potential in oncotherapy models [45] and are beginning to be utilized in CRISPR system delivery [46]. In the present study, CRISPRa components were delivered using electroporation and transfection, which are commonly used for efficient gene modulation in vitro. However, the delivery efficiency of these approaches remains limited, especially for in vivo applications [47].

Achieving cell-type-specific targeting remains a critical challenge for CRISPRa-based therapeutic applications. In the context of T cells, several complementary strategies can be considered, including the use of cell-specific promoters, receptor-targeted delivery systems, and ex vivo modification, followed by adoptive transfer [48,49,50]. Viral vector pseudotyping can be employed to enhance selective targeting of immune cell populations [51]. In addition, emerging non-viral strategies such as antibody-directed lipid nanoparticles targeting T cell surface markers (for example, CD3 and CD7) have demonstrated promising potential for selective in vivo delivery to T cell populations. Dual-targeting approaches (CD3/CD7) have been shown to enhance delivery efficiency across both resting and activated T cells, highlighting their potential [52]. In the context of *CTLA4* CRISPRa, a potential strategy would be to develop a vector system bearing dCas9-VPR under the control of the endogenous *CTLA4* promoter. This strategy could facilitate a more controlled gene activation in a physiological context, minimizing off-target effects and maximizing the therapeutic potential. Also, the integration of such a functional cassette into the target genome via a lentiviral vector would allow for long-term *CTLA4* activation in a physiological context. The duration of therapeutic effect represents an important issue of CRISPRa. Overall, CRISPRa activation peaks at 24–48 h, declines at 48–72 h, and ceases completely at 96 h post-electroporation [53]. In the current study, we observed the persistent presence of *CTLA4* protein for 72 h after electroporation. These results demonstrate the durability of the CRISPRa activation effect.

Another important issue that needs to be addressed when developing CRISPR-based protocols is the potential immunogenicity of the utilized components [54]. Indeed, pre-existing anti-SaCas9 and anti-SpCas9 antibodies [55], as well as anti-RfxCas13d antibodies [56], were described in a general population. Interestingly, the estimated percentage of positive pre-existing anti-CRISPR immunity varies across studies between 2.5 and 5% [55,57] and 58 and 95% [56,58]. Such variability may be explained by peculiarities of research methods and study design. Apart from pre-existing immunity, the CRISPR-based therapy can induce an immune response in naïve individuals upon administration [59,60]. The CRISPR immunogenicity represents an important issue hindering clinical implementation of CRISPR-based technologies. This problem is currently being addressed by rational design of minimally immunogenic CRISPR nucleases, which avoids potentially immunogenic epitopes of clinically relevant CRISPR nucleases [61]. As for the current study, further development of our CRISPRa approach requires assessing the immunogenicity of the protocol and then, if necessary, applying a rational design.

## 5. Conclusions

In the present study, we demonstrated that the CRISPRa system can effectively activate endogenous *CTLA4* expression to levels comparable to activated T cells, including models of *CTLA4* haploinsufficiency, even when conventional stimulation is insufficient. This suggests that this approach may overcome limitations associated with insufficient endogenous *CTLA4* expression under standard activation conditions. These fundings highlight the potential of CRISPRa as a tool for controlled modulation of *CTLA4* expression.

However, several limitations remain. Although no substantial off-target transcriptomic changes were detected in Jurkat cells, indicating a high level of specificity, further studies are required to assess specificity and safety in primary cells and in vivo systems. As this study was conducted on a Jurkat cell model, additional validation in primary human T cells is required.

Further research should focus on validating the functional effects of CRISPRa-induced *CTLA4* expression and on developing efficient and targeted delivery strategies for primary immune cells. It will also be important to evaluate the functional activity of CRISPRa-induced *CTLA4* protein, including its surface expression and ability to mediate *CTLA4*-dependent transendocytosis.

Overall, this proposed approach may serve as a basis for further development of CRISPRa-based strategies to restore *CTLA4* expression in immune dysregulation disorders, although additional validation is required before clinical translation.

In summary, we believe that the current study once again confirms the potential of the CRISPR platform for modulation of genome activity. However, it is also important to note that further progress of CRISPR-based technologies should not ignore the ethical issues that arise with the genome editing approached in general and CRISPR in particular. These include proper addressing of the safety risks, clear risk assessments in informed consents for patients before therapy, clear development of legislative acts in the field of genome editing, etc. [62,63].

## Figures and Tables

**Table 1:** List of gRNA sequences and adjacent PAM sites used for *CTLA4* CRISPR KO.

gRNA			
Region	Genomic Location (GRCh38)	Number	Sequence, 5′-3′; PAM
5′-end	chr2: 203,867,868–203,867,868	gRNA_1	TTTGAACCCACACAGAATCA; AGG
	chr2: 203,867,875–203,867,894	gRNA_3	CAAAGCTTCAGGATCCTGAA; AGG
3′-end	chr2: 203,873,518–203,873,537	gRNA_5	AATACACCAGGTAGAACACA; AGG

**Table 2:** List of primer sequences employed for genomic deletion screen/validation.

Amplicon Name	Oligo Direction	Sequence, 5′-3′
5′_CTLA4_flank	Forward	AAGTGCCTTCTGTGTGTGC
	Reverse	GTCCTGGTAGCCAGGTTC
CTLA4_deletion	Forward	ACAGCTAAACCCACGGCTTC
	Reverse	AACTCAGATACCACCAGCTG

**Table 3:** List of gRNA sequences applied in the epigenetic activation of the *CTLA4* gene.

gRNA	Oligo Name	Sequence, 5′-3′; PAM
1	CTLA4_A_gR1	TCTCTTAACCAAATGCTAAA; TGG
2	CTLA4_A_gR2	AGTTAGCAGCCTAGTAGTTT; TGG
3	CTLA4_A_gR3	TTTCTGAGCCCTTGGGCTAA; TGG
4	CTLA4_A_gR4	AAATCCTGCCATTAGCCCAA; GGG
5	CTLA4_A_gR5	TCAAGGGACCATTAGAAGGA; TGG
6	CTLA4_A_gR6	TGAATTGGACTGGATGGTTA; AGG
7	CTLA4_A_gR7	TAGGAGGACCCTTGTACTCC; AGG
8	CTLA4_A_gR8	TGAGCCCTTGGGCTAATGGC; AGG
9	CTLA4_A_gR9	ACCAAATGCTAAATGGATTT; AGG
10	CTLA4_A_gR10	TACATTTTCCATCCATGGAT; TGG

**Table 4:** The primers utilized for the assessment of *CTLA4* gene expression changes by qPCR.

Amplicon Name	Oligo Direction	Sequence, 5′-3′
CTLA4_3	Forward	GCAAGGTGGAGCTCATGTAC
	Reverse	AGACCCCTGTTGTAAGAGGG
RPL32_H	Forward	TCTCCTTCTCGGCATCATGG
	Reverse	CGAACCCTGTTGTCAATGCC
GAPDH_RT	Forward	CTCCTCTGACTTCAACAGCG
	Reverse	GCTGTAGCCAAATTCGTTGTC

**Table 5:** The percentage of *CTLA4*-positive cells upon the CRISPRa protocol in Jurkat*^CTLA4^*^+/+^, Jurkat*^CTLA4^*^+/−^, and Jurkat^*CTLA4*−/−^ cells compared to the corresponding negative controls.

Genotype	Replicate 1 (%)	Replicate 2 (%)	Replicate 3 (%)	% *CTLA4*^+^ Cells (Mean ± SD)
Jurkat*^CTLA4^*^+/+^	30.22	6.04	39.10	25.12 ± 17.10
Jurkat*^CTLA4^*^+/−^	5.55	2.53	7.82	5.3 ± 2.65
Jurkat^*CTLA4*−/−^	0.00	0.00	0.01	0.003 ± 0.005

## Data Availability

Research data are available upon request.

## Appendix A

**Table A1:** Primer characteristics and qPCR efficiency.

Gene	Amplification Size (bp)	Slope	R^2^	Efficiency (%)	Tm (°C)	Amplification Verification
*GAPDH*	126	−3.297	0.983	101	84.50	single peak
*RPL32*	153	−3.438	0.990	95.4	84.00	single peak
*CTLA4*	214	−3.582	0.982	90.2	83.50	single peak

**Table A2:** Oligonucleotide primers and predicted amplicon sizes for integrity PCR of the 10-gRNA expression plasmid.

Amplicon Name	Primer Name	Primer Sequence, 5′-3′	Amplicon Size
PCR_1	M13_fw	GTAAAACGACGGCCAGT	458 bp
	Barcode_1_R	TGGGAAATAGGCCCTCCATG	
PCR_2	CTLA4_gRNA_1	GTCTCTTAACCAAATGCTAAA	535 bp
	Barcode_2_R	TGGGAAATAGGCCCTCGTCC	
PCR_3	CTLA4_gRNA_2	GAGTTAGCAGCCTAGTAGTTT	535 bp
	Barcode_3_R	TGGGAAATAGGCCCTCCTGG	
PCR_4	CTLA4_gRNA_3	GTTTCTGAGCCCTTGGGCTAA	535 bp
	Barcode_4_R	TGGGAAATAGGCCCTCAACA	
PCR_5	CTLA4_gRNA_4	GAAATCCTGCCATTAGCCCAA	535 bp
	Barcode_5_R	TGGGAAATAGGCCCTCTGCA	
PCR_6	CTLA4_gRNA_5	GAAATCCTGCCATTAGCCCAA	535 bp
	Barcode_6_R	TGGGAAATAGGCCCTCACCG	
PCR_7	CTLA4_gRNA_6	GAAATCCTGCCATTAGCCCAA	535 bp
	Barcode_7_R	TGGGAAATAGGCCCTCTTTC	
PCR_8	CTLA4_gRNA_7	GAAATCCTGCCATTAGCCCAA	535 bp
	Barcode_8_R	TGGGAAATAGGCCCTCTCGA	
PCR_9	CTLA4_gRNA_8	GAAATCCTGCCATTAGCCCAA	535 bp
	Barcode_9_R	TGGGAAATAGGCCCTCGAGC	
PCR_10	CTLA4_gRNA_9	GAAATCCTGCCATTAGCCCAA	538 bp
	FUS_AvrII_rev	GTTTCGAGCGCTTGCTTG	

**Table A3:** Pairwise comparations of % *CTLA4*+ cells among Jurkat genotypes.

Comparison	Adjusted *p*-Value
Jurkat*^CTLA4^*^+/+^ vs. Jurkat*^CTLA4^*^+/−^	0.8841
Jurkat*^CTLA4^*^+/+^ vs. Jurkat^*CTLA4*−/−^	0.0328
Jurkat*^CTLA4^*^+/−^ vs. Jurkat^*CTLA4*−/−^	0.4032

Statistical analysis was performed as described in the Statistical Methods section.
